# Supplementary material for: Development of a skin- and neuro-attenuated live vaccine for varicella
Source: Nat Commun. 2022 Feb 11;13:824. doi: 10.1038/s41467-022-28329-1 (PMC8837607; doi:10.1038/s41467-022-28329-1)
Supplement: Supplementary file 1 — Supplementary Information [file 41467_2022_28329_MOESM1_ESM.pdf]

Supplementary Information for

Development of a Skin- and Neuro-Attenuated Live Vaccine for Varicella

Wei Wang,[1*] Dequan Pan,[1*] Wenkun Fu,[1*] Xiangzhong Ye,[3*] Jinle Han,[3] Lianwei Yang,[3] Jizong Jia,[3] Jian Liu,[1] Rui Zhu,[1] Yali Zhang,[1] Che Liu,[1] Jianghui Ye,[1] Anca Selariu,[2] Yuqiong Que,[1] Qinjian Zhao,[1] Ting Wu,[1] Yimin Li,[3] Jun Zhang,[1†] Tong Cheng, [1†] Hua Zhu,[2†] Ningshao Xia[1†]

[*]These authors contributed equally to this work.
[†]Correspondence should be addressed to N.X. (nsxia@xmu.edu.cn), H.Z.(zhuhu@njms.rutgers.edu), T.C. (tcheng@xmu.edu.cn) or J.Z. (zhangj@xmu.edu.cn)

This PDF file includes:

Supplementary Figures 1-14
Supplementary Tables 1-7

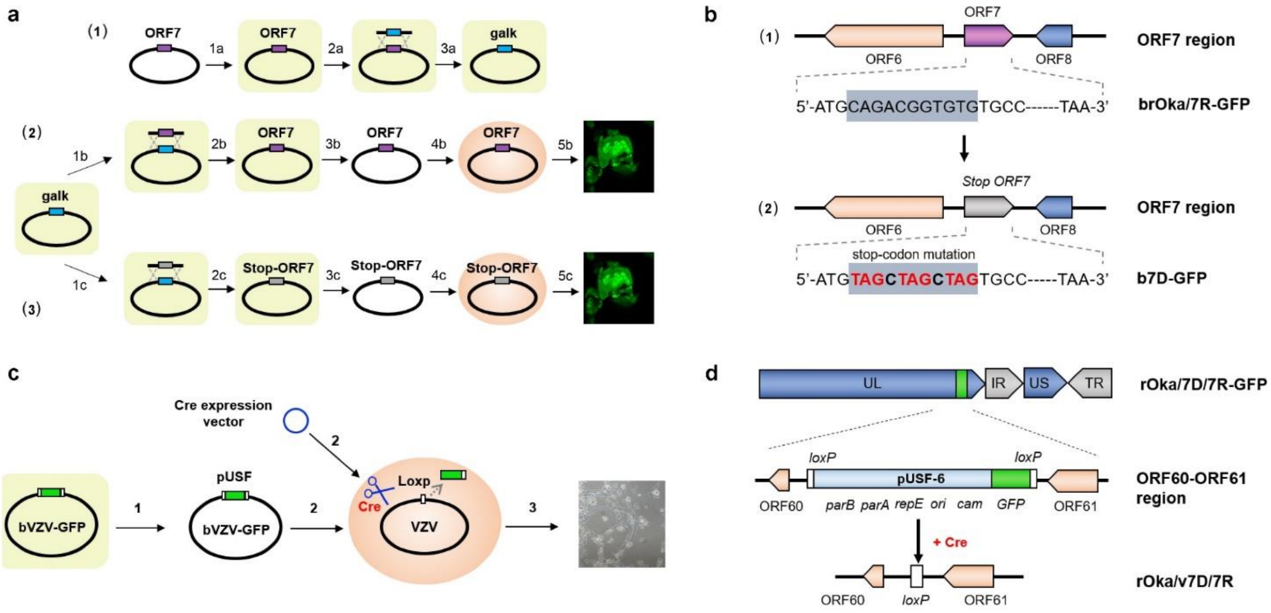

Supplementary Fig. 1. Schematic diagram on the construction of v7D. **a** Generation of the VZV 7D- and 7R-GFP BAC clones from the VZV rOka-GFP BAC clone. (1) ORF7 in the VZV rOka-GFP BAC clone (brOka-GFP) was replaced by a galk gene to generate an ORF7 deletion mutant BAC clone. (2) The revertant VZV BAC, b7R-GFP was made by rescuing the wild-type ORF7 gene back into the ORF7-deleted VZV BAC. (3) The VZV 7D-GFP BAC clone, b7D-GFP was made by replacing the galk gene with the ORF7 gene containing stop codons at the 5` end. **b** Compared to brOka/7R-GFP, in b7D-GFP, the 11-bp region downstream of the ATG start codon of ORF7 was mutated into a three-frame stop codon cassette. **c, d** Generation of the ORF7-deficient candidate vaccine virus v7D, the wild-type virus rOka and the rescue virus 7R. Viruses were reconstituted from VZV BAC DNA, and were further passaged in the MRC-5 cells expressing Cre recombinase to excise the BAC vector sequences. The yellow rectangle represents *E. coli* with a recombination system. The orange ellipse represents MRC-5 cells.

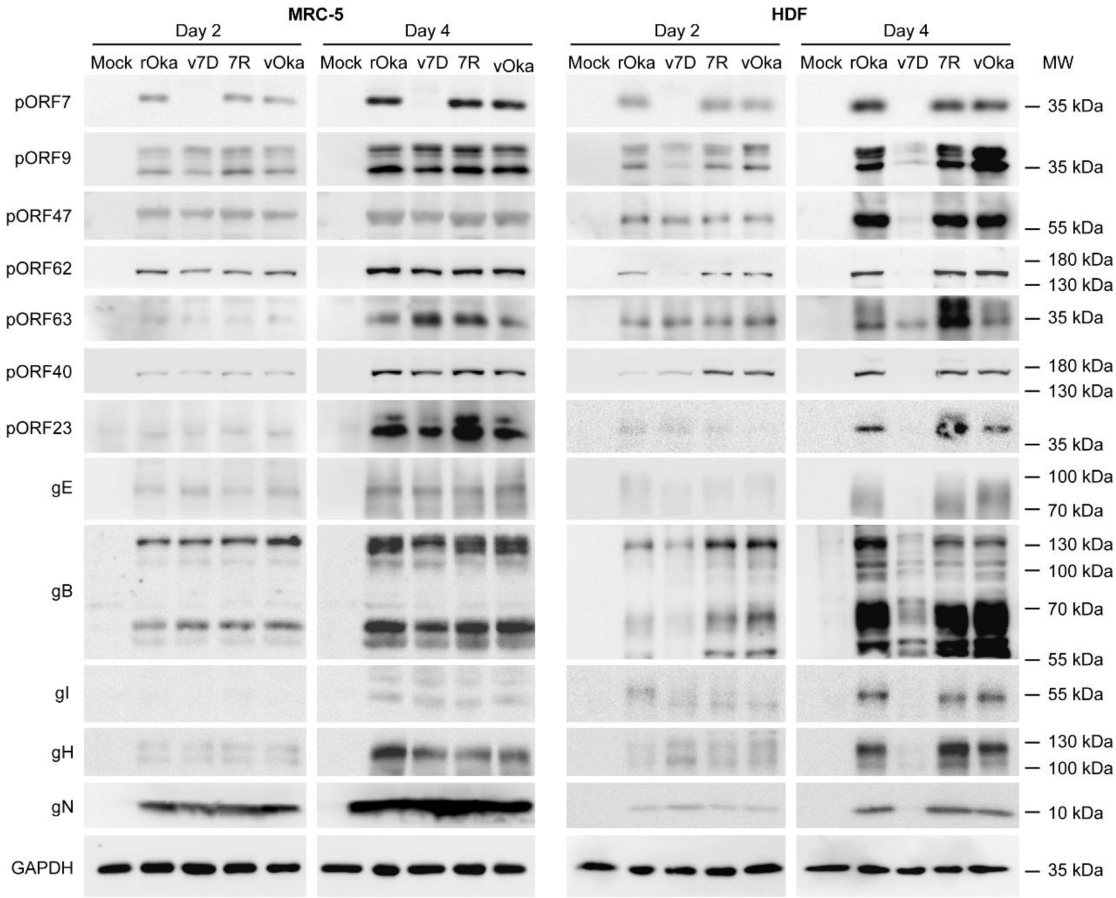
MRC-5
HDF
Day 2
Day 4
Mock rOka v7D 7R vOka
MW
pORF7
pORF9
pORF47
pORF62
pORF63
pORF40
pORF23
gE
gB
gI
gH
gN
GAPDH
35 kDa
55 kDa
180 kDa
130 kDa
100 kDa
70 kDa
10 kDa

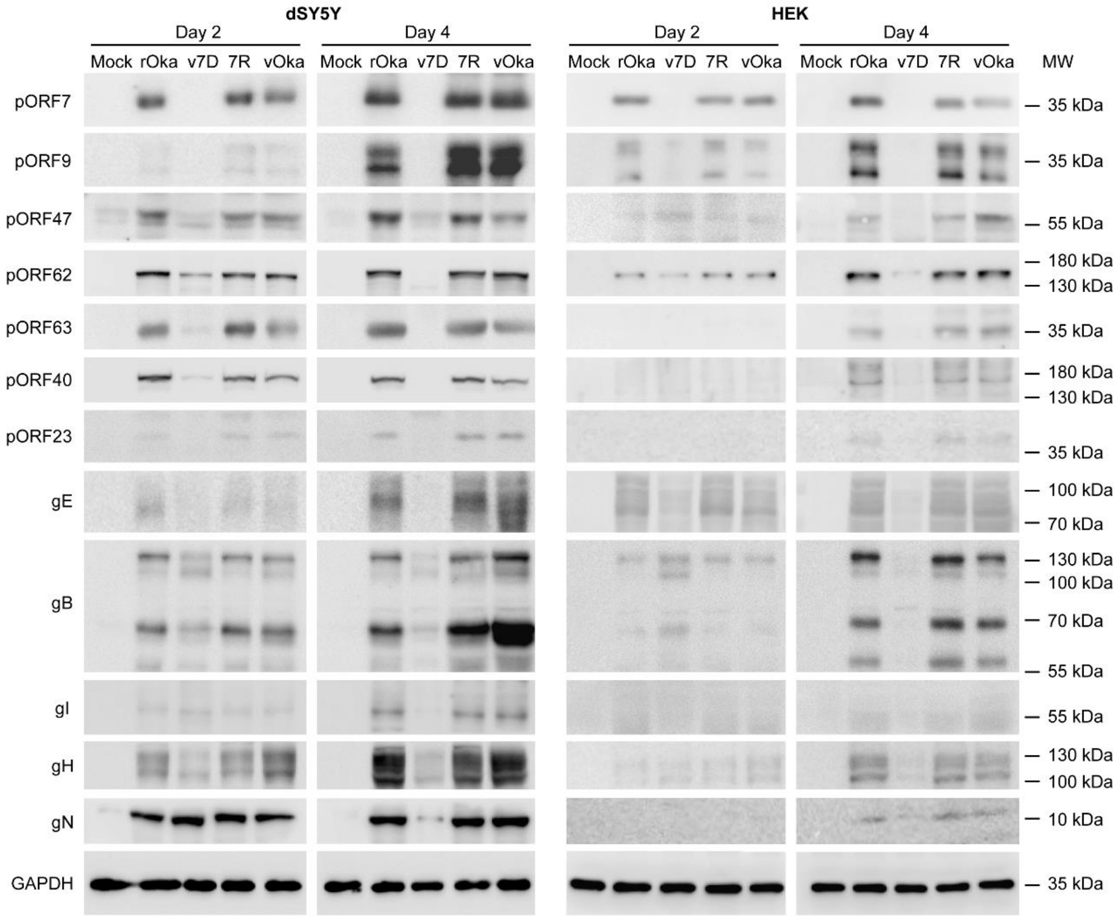
dSY5Y
HEK
Day 2
Day 4
Mock rOka v7D 7R vOka
MW
pORF7
pORF9
pORF47
pORF62
pORF63
pORF40
pORF23
gE
gB
gI
gH
gN
GAPDH
35 kDa
55 kDa
180 kDa
130 kDa
100 kDa
70 kDa
10 kDa

Supplementary Fig. 2. Expression of typical viral proteins in different v7D-infected cell types in Fig. 1. MRC-5 cells, dSY5Y cells, HDFs and HEKs were mock-infected or infected with cell-free rOka, v7D, 7R and vOka, respectively. For MRC-5 cells, dSY5Y cells and HDFs, MOI=0.01. For HEKs, MOI=0.2. Cell lysates were prepared from the harvested at 2 and 4 dpi and tested by Western blot for 12 VZV structural proteins, including capsid proteins (pORF23 and pORF40), tegument proteins (pORF7, pORF9, pORF47, pORF62 and pORF63) and glycoproteins (gE, gB, gH, gI and gN). The experiments were repeated twice, and representative figures are shown. Source data are provided as a Source Data file.

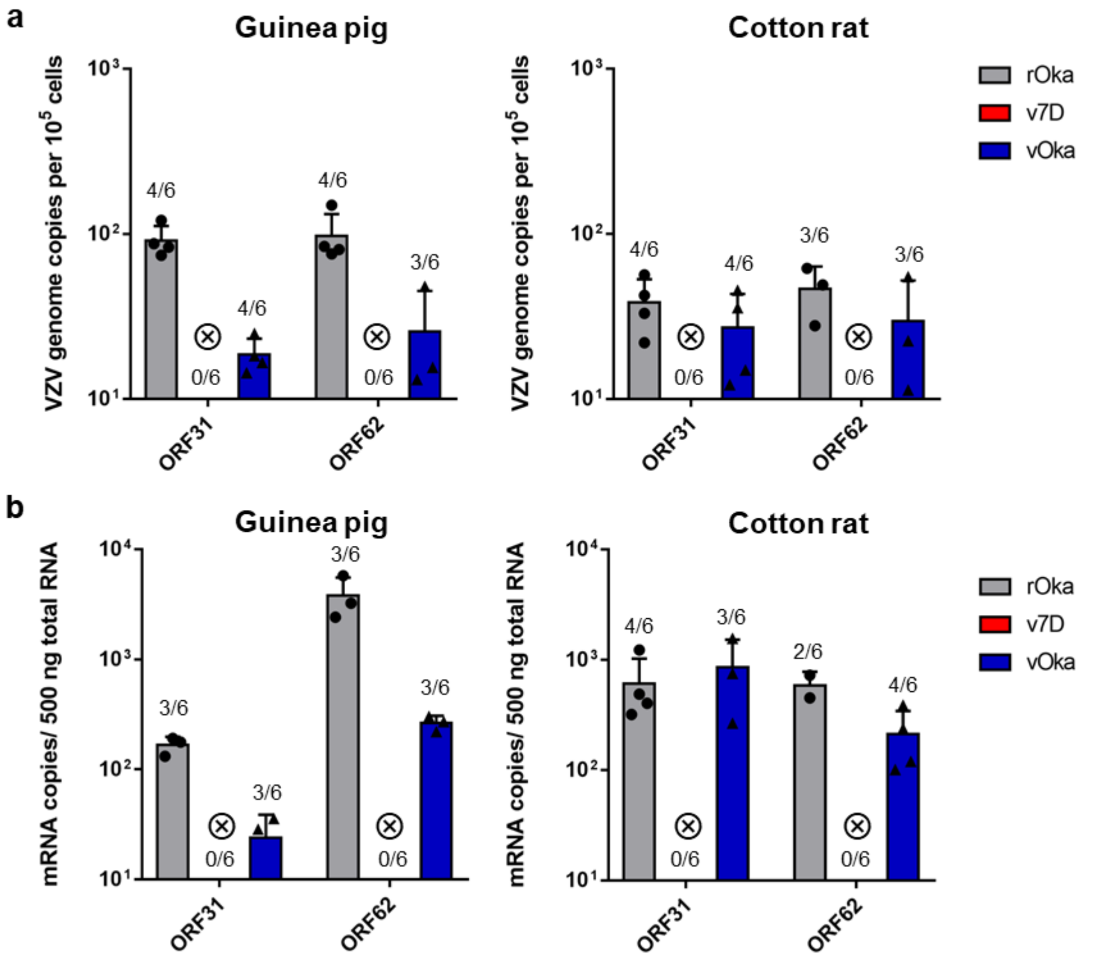

Supplementary Fig. 3. Determination of (**a**) VZV genome and (**b**) mRNA copy numbers in DRGs from infected guinea pigs and cotton rats using probes for ORF31 and ORF62. The number of animals from which VZV DNA or RNA was detected in DRGs per number of VZV-inoculated animals per group is shown above each bar. Mean copy numbers per group were calculated based on data from animals that had detectable VZV DNA or RNA in their DRGs. A circle containing a cross indicates that none of the six animals in the v7D group had detectable VZV DNA or RNA. The results are represented as averages ± the SD. The experiments were repeated twice, and representative results are shown. Source data are provided as a Source Data file.

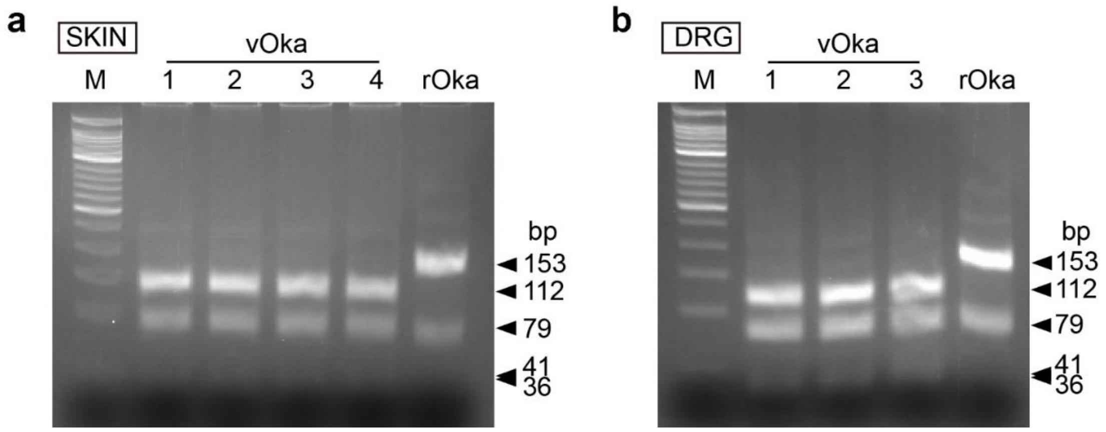

Supplementary Fig. 4. Verification of vOka infection in human skin and DRG xenografts by PCR-RFLP analysis in Fig. 3. The 268-bp PCR amplicon of VZV ORF62 was digested into 112-, 79-, 41-, and 36-bp SmaI fragments for vOka virus isolated from the inoculated (**a**) skin (lane 1 to 4) and (**b**) DRG xenografts (lane 1 to 3). For the wild-type rOka virus (The rightmost lane in **a** and **b**), the amplicon was digested into 153-, 79-, and 36-bp SmaI fragments. The experiments were repeated twice with similar results. Lane M, molecular size marker set (2500 bp + 50 to 800 bp in 50-bp multiples). Source data are provided as a Source Data file.

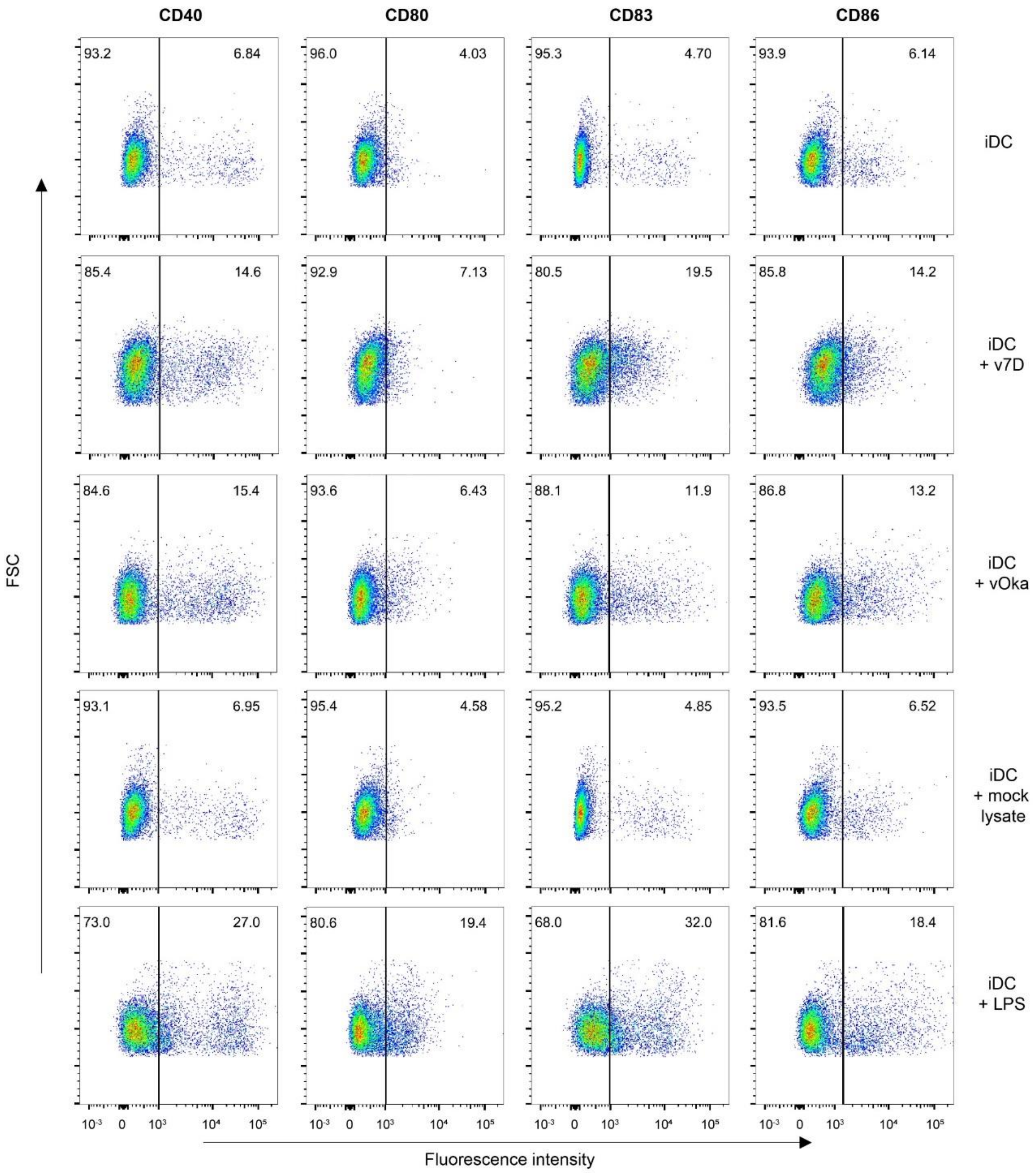

Supplementary Fig. 5. Flow cytometry analysis of DC activation/maturation phenotype in Fig. 4. Representative flow cytometry data for CD40, CD80, CD83 and CD86 positive DCs shown in Fig. 4c. Untreated iDCs and lipopolysaccharide (LPS; 100ng/ml)–treated DCs were used as negative and positive controls, respectively. Source data are provided as a Source Data file.

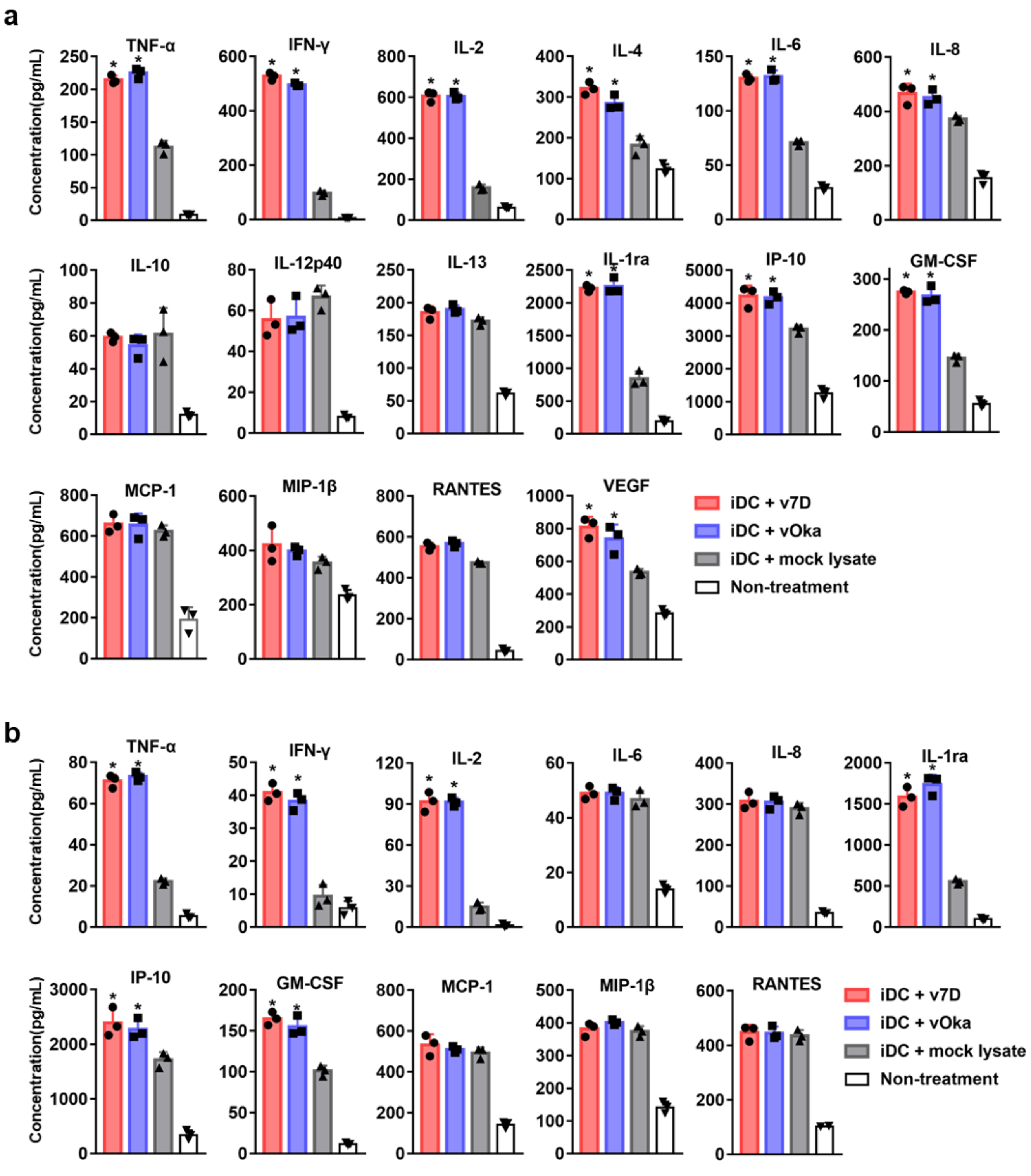

Supplementary Fig. 6. Cytokine/chemokine analysis of the DC-T-cell co-culture supernatants. A total of 16 and 11 cytokines/chemokines were found to be increased in the supernatants of (**a**) DC:CD4^{+} T cell and (**b**) DC:CD8^{+} T cell co-cultures, respectively, at day 5. The results are represented as averages ± the SD (n = 3 per group). Asterisks denote a significant difference ($p<0.05$) compared to the mock-treated controls as determined by one-way ANOVA with Tukey's post-hoc test. Detailed information about the statistics and values are provided in Supplementary Table 3. Source data are provided as a Source Data file.

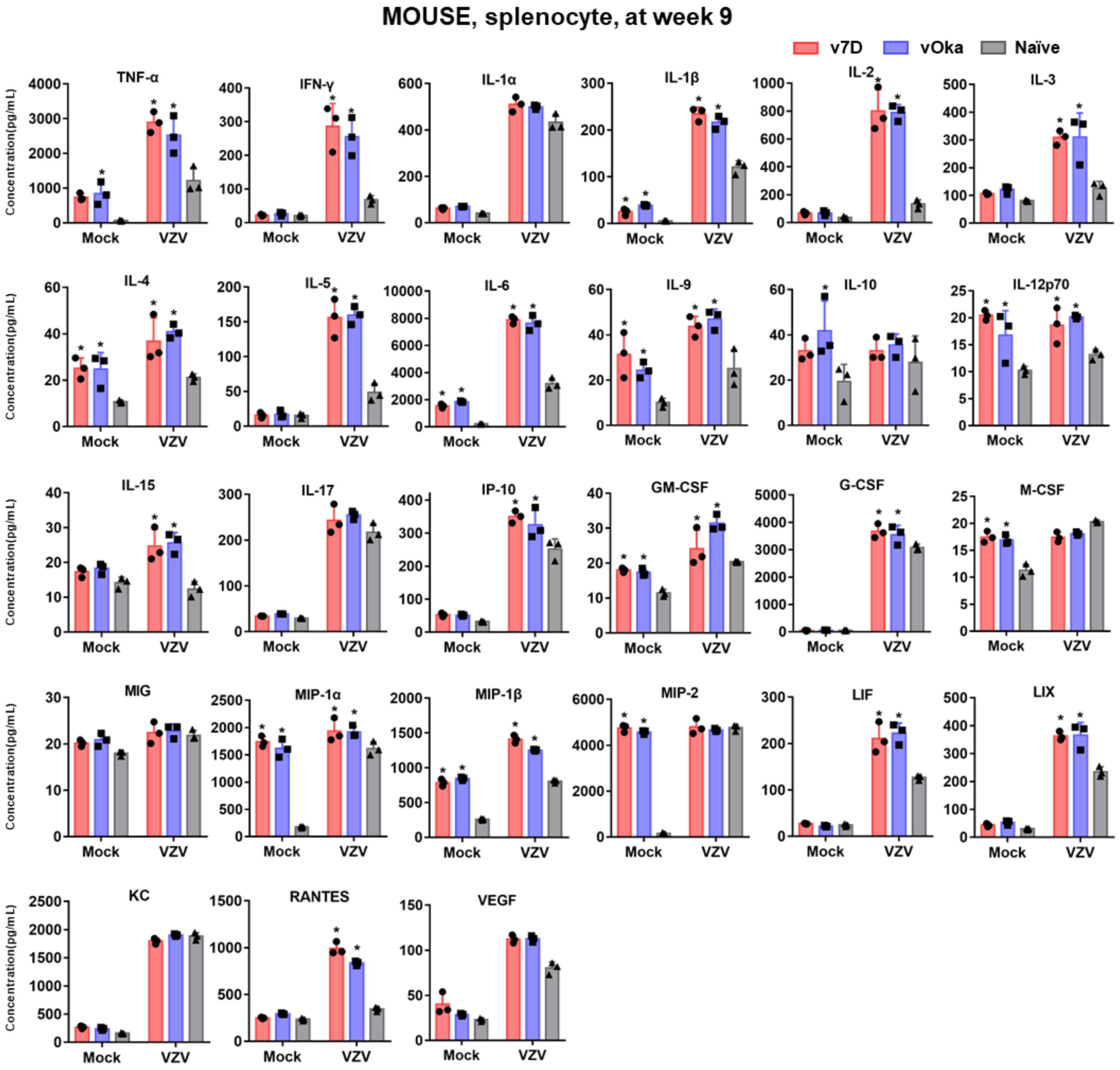

Supplementary Fig. 7. Analysis of cytokine/chemokine responses to VZV antigens by splenocytes from v7D-immunized mice in Fig. 5. Three weeks after the final immunization (week 9), a total of 27 cytokines/chemokines were found to be increased in the supernatants of cultured splenocytes from v7D- and vOka-immunized mice (n = 3 per group) in response to stimulation with rOka virus. The results are represented as averages ± the SD. Asterisks denote a significant difference ($p<0.05$) compared to the untreated naïve controls as determined by two-way ANOVA with Tukey's post-hoc test. Detailed information about the statistics and values are provided in Supplementary Table 3. Source data are provided as a Source Data file.

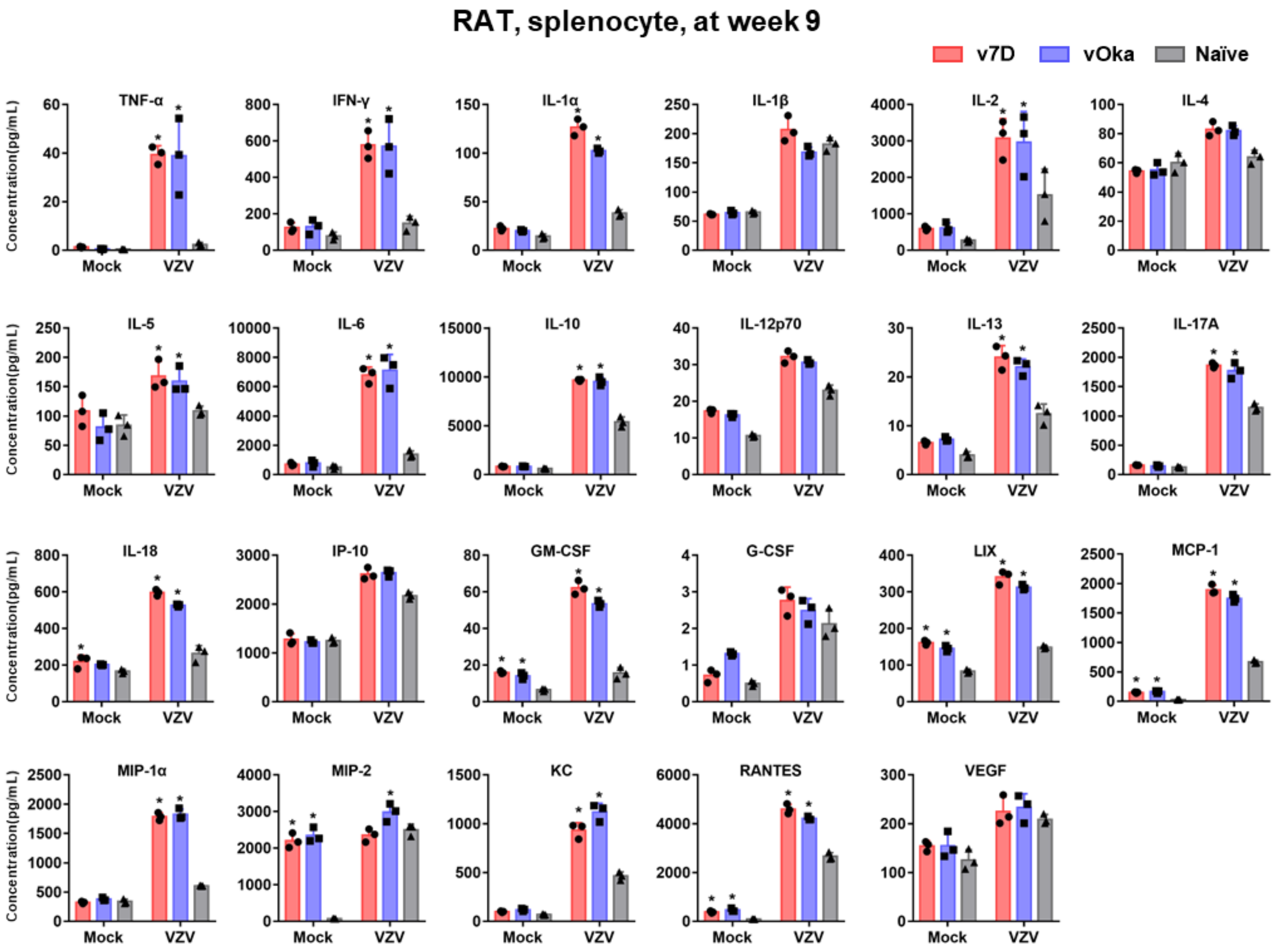

Supplementary Fig. 8. Analysis of cytokine/chemokine responses to VZV antigens by splenocytes from v7D-immunized rats in Fig. 5. Three weeks after the final immunization (week 9), a total of 23 cytokines/chemokines were found to be increased in the supernatants of cultured splenocytes from v7D- and vOka-immunized rats (n = 3 per group) in response to stimulation with rOka virus. The results are represented as averages ± the SD. Asterisks denote a significant difference ($p<0.05$) compared to the untreated naïve controls as determined by two-way ANOVA with Tukey's post-hoc test. Detailed information about the statistics and values are provided in Supplementary Table 3. Source data are provided as a Source Data file.

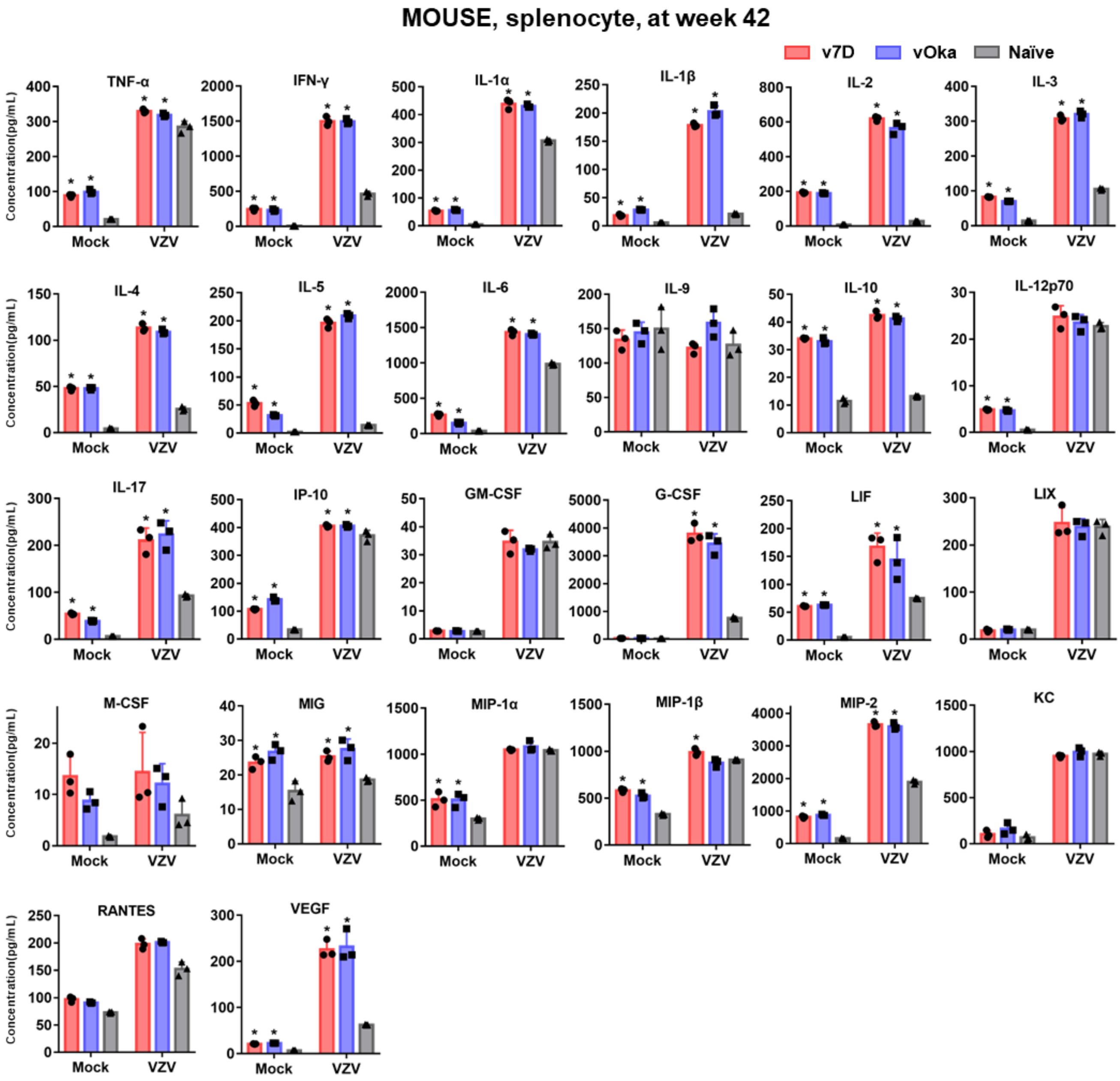

Supplementary Fig. 9. Analysis of cytokine/chemokine responses to VZV antigens by splenocytes from v7D-immunized mice at the end of this study (week 42). A total of 26 cytokines/chemokines were found to be increased in the supernatants of cultured splenocytes from v7D- and vOka-immunized mice (n = 3 per group) in response to stimulation with rOka virus. The results are represented as averages ± the SD. Asterisks denote a significant difference ($p<0.05$) compared to the untreated naïve controls as determined by two-way ANOVA with Tukey's post-hoc test. Detailed information about the statistics and values are provided in Supplementary Table 3. Source data are provided as a Source Data file.

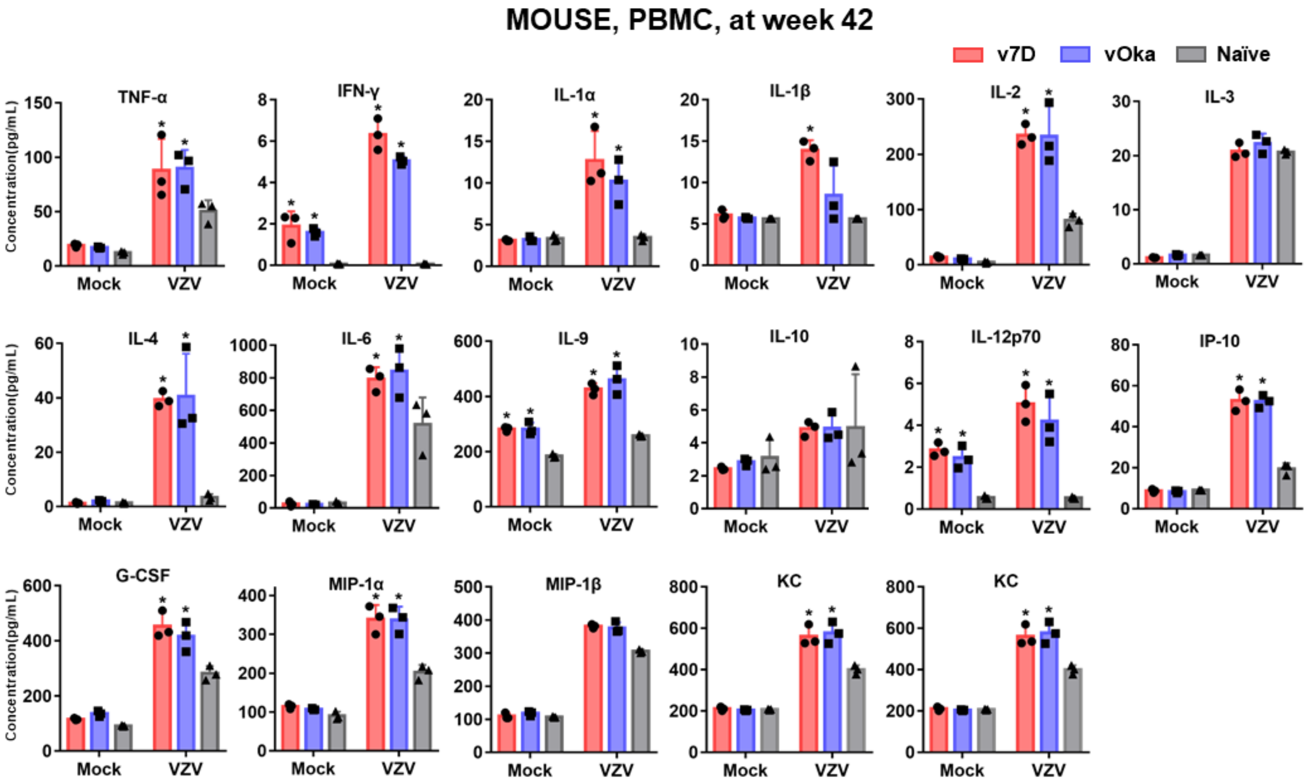

Supplementary Fig. 10. Analysis of cytokine/chemokine responses to VZV antigens by PBMCs from v7D-immunized mice at the end of this study (week 42). A total of 17 cytokines/chemokines were found to be increased in the supernatants of cultured PBMCs from v7D- and vOka-immunized mice (n = 3 per group) in response to stimulation with rOka virus. The results are represented as averages ± the SD. Asterisks denote a significant difference ($p<0.05$) compared to the untreated naïve controls as determined by two-way ANOVA with Tukey's post-hoc test. Detailed information about the statistics and values are provided in Supplementary Table 3. Source data are provided as a Source Data file.

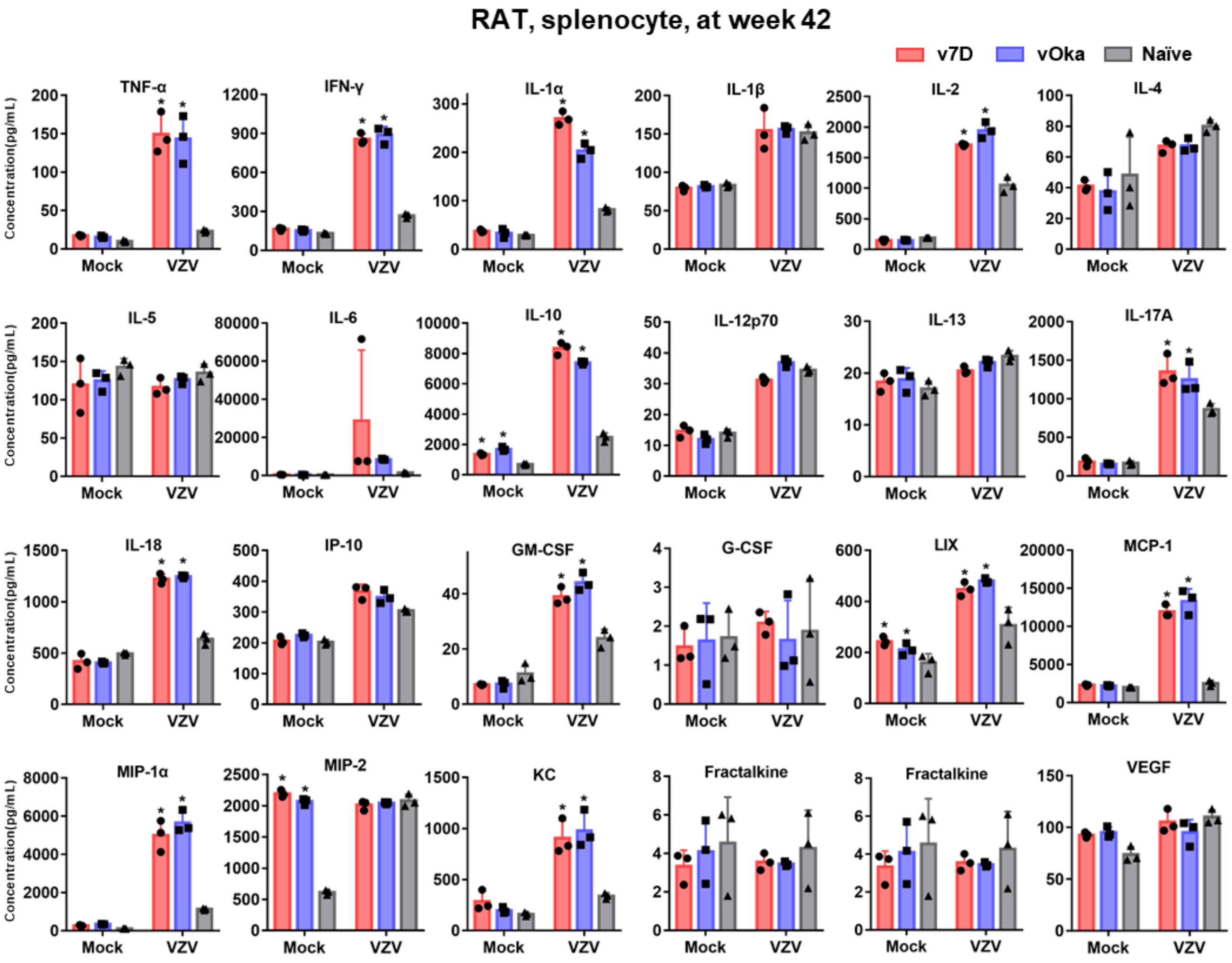

Supplementary Fig. 11. Analysis of cytokine/chemokine responses to VZV antigens by splenocytes from v7D-immunized rats at the end of this study (week 42). A total of 24 cytokines/chemokines were found to be increased in the supernatants of cultured splenocytes from v7D- and vOka-immunized rats (n = 3 per group) in response to stimulation with rOka virus. The results are represented as averages ± the SD. Asterisks denote a significant difference ($p<0.05$) compared to the untreated naïve controls as determined by two-way ANOVA with Tukey's post-hoc test. Detailed information about the statistics and values are provided in Supplementary Table 3. Source data are provided as a Source Data file.

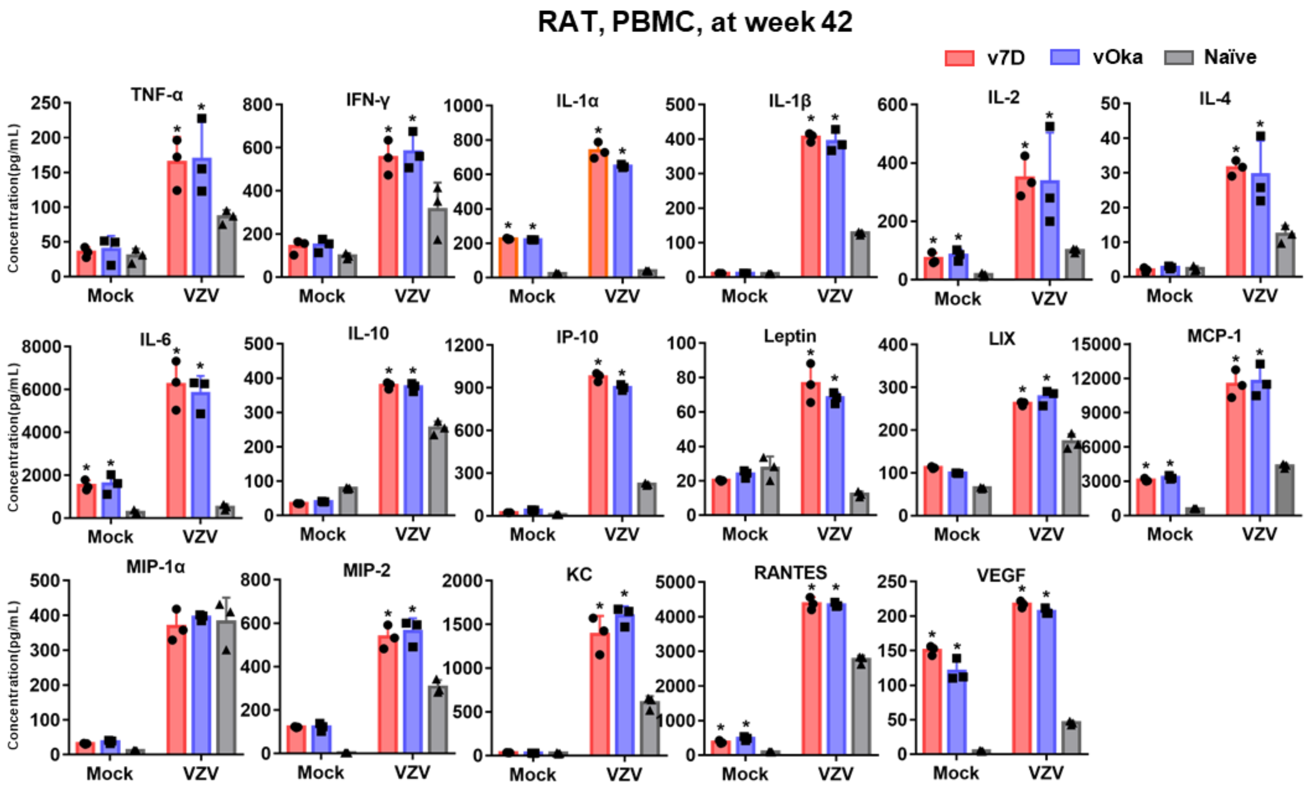

Supplementary Fig. 12. Analysis of cytokine/chemokine responses to VZV antigens by PBMCs from v7D-immunized rats at the end of this study (week 42). A total of 17 cytokines/chemokines were found to be increased in the supernatants of cultured PBMCs from v7D- and vOka-immunized rats (n = 3 per group) in response to stimulation with rOka virus. The results are represented as averages ± the SD. Asterisks denote a significant difference ($p<0.05$) compared to the untreated naïve controls as determined by two-way ANOVA with Tukey's post-hoc test. Detailed information about the statistics and values are provided in Supplementary Table 3. Source data are provided as a Source Data file.

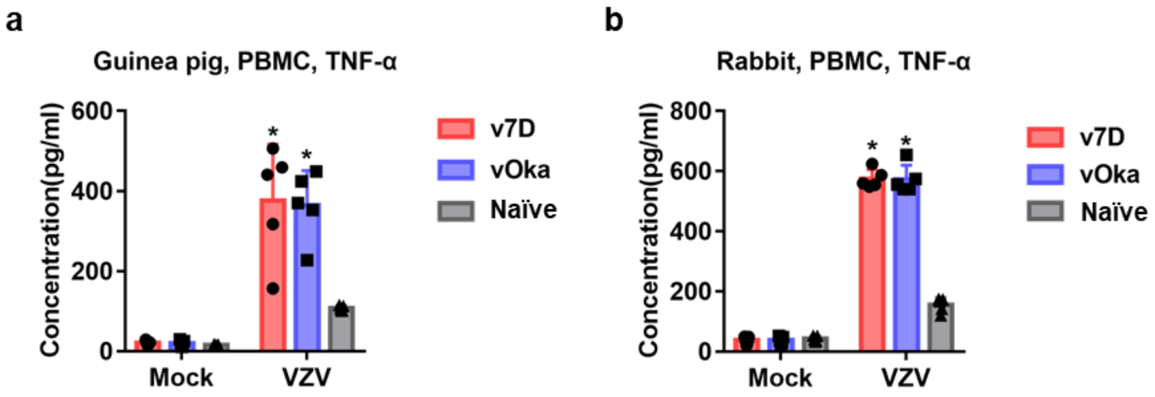

Supplementary Fig. 13. Analysis of cytokine/chemokine responses to VZV antigens by PBMCs from v7D-immunized guinea pigs and rabbits at the end of this study (week 42). TNF-α was determined in the supernatants of cultured PBMCs from v7D- and vOka-immunized (**a**) guinea pigs and (**b**) rabbits (n = 3 per group) in response to stimulation with rOka virus. The results are represented as averages ± the SD. Asterisks denote a significant difference ($p<0.05$) compared to the untreated naïve controls as determined by two-way ANOVA with Tukey's post-hoc test. Detailed information about the statistics and values are provided in Supplementary Table 3. Source data are provided as a Source Data file.

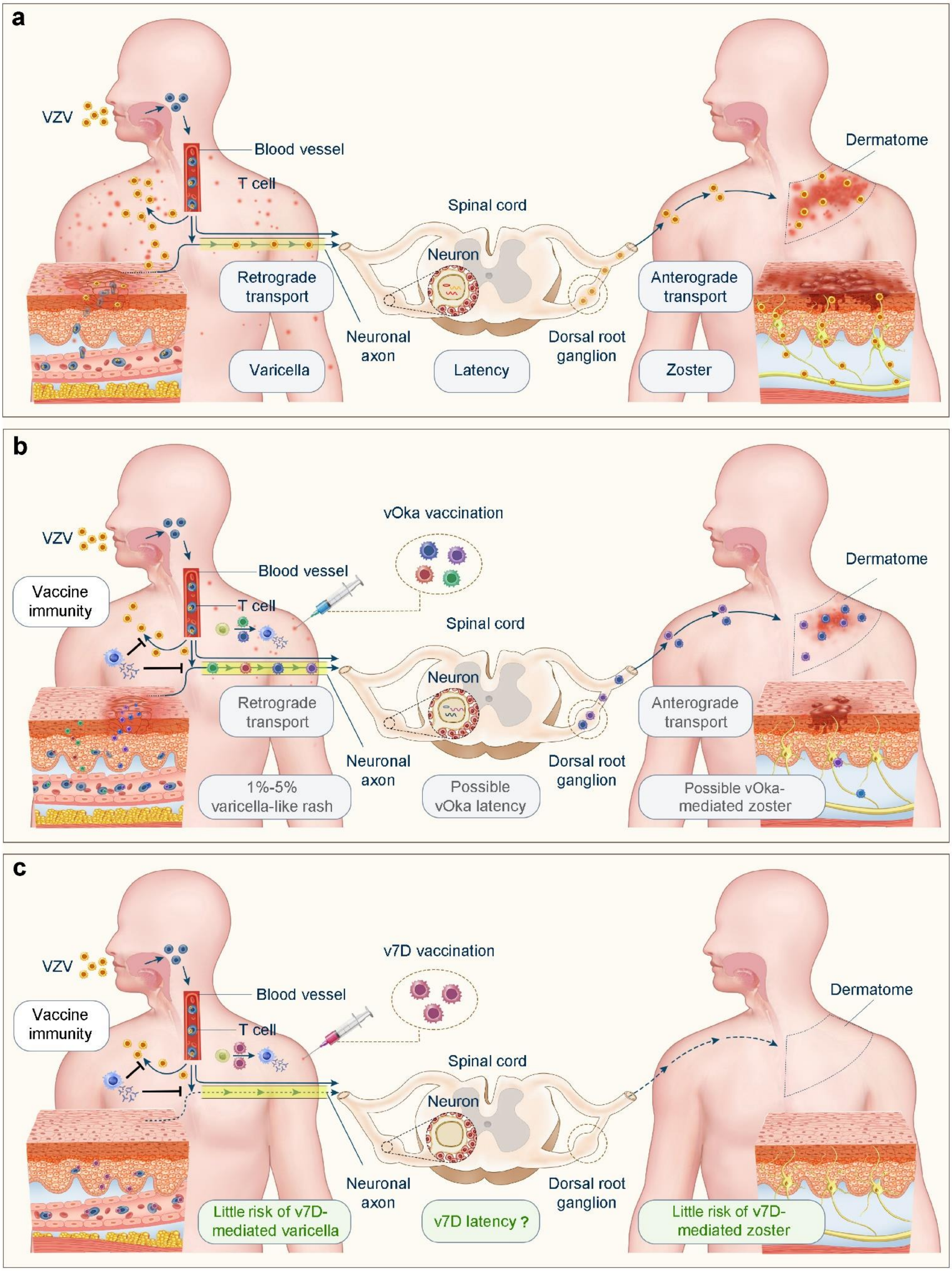
a
VZV
Blood vessel
T cell
Spinal cord
Neuron
Retrograde transport
Neuronal axon
Varicella
Latency
Dorsal root ganglion
Anterograde transport
Zoster
Dermatome
b
VZV
Vaccine immunity
Blood vessel
T cell
vOka vaccination
Spinal cord
Neuron
Retrograde transport
Neuronal axon
1%-5% varicella-like rash
Possible vOka latency
Dorsal root ganglion
Anterograde transport
Possible vOka-mediated zoster
Dermatome
c
VZV
Vaccine immunity
Blood vessel
T cell
v7D vaccination
Spinal cord
Neuron
Neuronal axon
Little risk of v7D-mediated varicella
v7D latency ?
Dorsal root ganglion
Little risk of v7D-mediated zoster
Dermatome

Supplementary Fig. 14. Hypothetical model showing potential safety and effectiveness of v7D vaccination in humans. **a** Pathogenesis and disease consequences of VZV infection. VZV infects the respiratory mucosal epithelium as a portal of entry and spreads to regional lymph nodes, where VZV infects T cells. The infected T cells deliver VZV to skin epithelial cells for replication through the circulating blood, causing crops of cutaneous vesicles. VZV establishes latency in dorsal root ganglia by retrograde transport along nerve axons or by viremia. Reactivation from latency returns VZV to the skin for replication via anterograde transport in axons, causing lesions in the dermatome that is innervated by the affected ganglia. **b** Adverse effects of vOka vaccination. vOka is a live-attenuated vaccine VZV strain consisting of several genetically distinct haplotypes (indicated by different colors). vOka elicits protective immunity against wild-type VZV infection, but may cause varicella-like rashes, establish latency and reactivate to cause vaccine-associated HZ in the recipients. However, no one vOka haplotype has been confirmed responsible for vaccine-associated diseases. **c** Potential safety and immunogenicity of v7D vaccination in humans. v7D is a pure, genetically-defined live-attenuated vaccine strain of VZV (deep pink). v7D lacks skin- and neuro-virulence and thus has little risk of varicella-like rash and vaccine-associated HZ. The capability of v7D to establish neuronal latency remains elusive. On the other hand, v7D retains lymphotropism for dissemination to elicit protective anti-VZV immune responses within the recipients.

Supplementary Table 1. Genome mutations after serial passages of v7D in cell cultures[†].

NO.	Position (POka)	Wild type	v7D				Location	Description
			P12	P15	P16	P25		
1	6	A	A	C	A	A	TRL[#]	Non-coding region
2	12	C	T	C	C	C	TRL	Non-coding region
3	16	C	G	G	G	C	TRL	Non-coding region
4	24	T	T	C	T	T	TRL	Non-coding region
5	8606-8632	ATGCAGACGGTGTGTGCCAGCTTATGT	ATGTAGCTAGCTAGTGCCAGCTTA	ATGTAGCTAGCTAGTGCCAGCTTA	ATGTAGCTAGCTAGTGCCAGCTTA	ATGTAGCTAGCTAGTGCCAGCTTA	ORF7	Three-frame stop codon
6	42282	PolyA	The same as wild type	The same as wild type	One more A	One more A	ORF22	Non-coding region
7	109740-110009	Ten repetitive sequences of 27 bp[a]	Two repetitive sequences of 27 bp	Three repetitive sequences of 27 bp	Two repetitive sequences of 27 bp	Three repetitive sequences of 27 bp	ORF62-ORF63	Non-coding region
8	110322	PolyTA	One TA missing	One more TA	One TA missing	One more TA	ORF62-ORF63	Non-coding region
9	112243	PolyA	Four more As	Four more As	Four more As	Four more As	ORF64	Non-coding region
10	117865	PolyT	Five Ts missing	Five Ts missing	Five Ts missing	Five Ts missing	ORF64	Non-coding region
11	120055	TTT	ND*	TTT	TTT	TTT	ORF70-ORF71	Non-coding region
12	120100-120369	Ten repetitive sequences of 27 bp[b]	Ten repetitive sequences of 27 bp	Ten repetitive sequences of 27 bp	Two repetitive sequences of 27 bp	Three repetitive sequences of 27 bp	ORF70-ORF71	Non-coding region

[†] Genomic DNA sequencing was performed by the Beijing Genomics Institute (BGI; Wuhan, China). The three-frame stop codon after the ATG codon of ORF7 was an experimental design to abrogate ORF7 expression in v7D. Other mutations were found only in the non-coding regions, including the polyA, polyTA, polyT and GC-rich regions, within the genome, and could be sequencing errors.

[#] TRL, terminal long repeats.

* ND, not detected.

[a] A 27-bp repetitive sequence: GGGGAGGGGGCGCGGTACCCCGCCGAT.

[b] A 27-bp repetitive sequence: CGGCGGGGTACCGCGCCCCCTCCCCAT.

Supplementary Table 2. Tissue distribution analysis by PCR for cynomolgus macaques in the repeated subcutaneous dose toxicity study†.

Sampling time point	*Animal NO.	Blood	Brain	Spleen	Liver	Kidney	Ovary/ Testis	Heart	Lung	DRG	Skin (mammary gland)	Skin (injection site)
Day 45	1514011	-	-	-	-	-	-	-	-	-	-	-
	1514012	-	-	-	-	-	-	-	-	-	-	-
	1514013	-	-	-	-	-	-	-	-	-	-	-
	1514016	-	-	-	-	-	-	-	-	-	-	-
	1514017	-	-	-	-	-	-	-	-	-	-	-
	1514018	-	-	-	-	-	-	-	-	-	-	-
	1514021	-	-	-	-	-	-	-	-	-	-	-
	1514022	-	-	-	-	-	-	-	-	-	-	+
	1514023	-	-	-	-	-	-	-	-	-	-	+
	1514026	-	-	-	-	-	-	-	-	-	-	+
	1514027	-	-	-	-	-	-	-	-	-	-	+
	1514028	-	-	-	-	-	-	-	-	-	-	-
	1514031	-	-	-	-	-	-	-	-	-	-	+
	1514032	-	-	-	-	-	-	-	-	-	-	+
	1514033	-	-	-	-	-	-	-	-	-	-	+
	1514036	-	-	-	-	-	-	-	-	-	-	+
	1514037	-	-	-	-	-	-	-	-	-	-	+
	1514038	-	-	-	-	-	-	-	-	-	-	+
Day 85	1514014	-	-	-	-	-	-	-	-	-	-	-
	1514015	-	-	-	-	-	-	-	-	-	-	-
	1514019	-	-	-	-	-	-	-	-	-	-	-
	1514020	-	-	-	-	-	-	-	-	-	-	-
	1514024	-	-	-	-	-	-	-	-	-	-	-
	1514025	-	-	-	-	-	-	-	-	-	-	-
	1514029	-	-	-	-	-	-	-	-	-	-	-
	1514030	-	-	-	-	-	-	-	-	-	-	-
	1514034	-	-	-	-	-	-	-	-	-	-	+
	1514035	-	-	-	-	-	-	-	-	-	-	+
	1514039	-	-	-	-	-	-	-	-	-	-	-
	1514040	-	-	-	-	-	-	-	-	-	-	-

† Total DNA was isolated from different tissues from six of the ten animals per group at day 45 and from the rest animals at day 85 for PCR analysis of VZV DNA. Source data are provided as a Source Data file.

*Group information. (1) The mock group (saline): Male, NO. 1514011-1514015; Female, NO. 1514016-1514020. (2) The v7D group 1 (1×10^4 PFU v7D/dose): Male, NO. 1514021-1514025; Female, NO. 1514026-1514030. (3) The v7D group 2 (5×10^4 PFU v7D/dose): Male, NO. 1514031-1514035; Female, NO. 1514036-1514040.

+ indicates PCR positive; - indicates PCR negative.

Supplementary Table 3. Results of statistical analysis.

Figure	Sample size (n)	Statistical Test	Values
2b	n=3 per group	One-way ANOVA with Tukey's post-hoc test	Mock vs rOka: p<0.0001<br>Mock vs v7D: p<0.0001<br>Mock vs vOka: p<0.0001
4b	n=3 per group	One-way ANOVA with Tukey's post-hoc test	Mock vs rOka: p<0.0001<br>Mock vs v7D: p<0.0001<br>Mock vs vOka: p<0.0001
4c	n=3 per group	Two-way ANOVA with Tukey's post-hoc test	CD40: iDC + mock lysate vs iDC +v7D: p<0.0001; iDC + mock lysate vs iDC +vOka: p<0.0001<br>CD80: iDC + mock lysate vs iDC +v7D: p=0.0085; iDC + mock lysate vs iDC +vOka: p=0.0779<br>CD83: iDC + mock lysate vs iDC +v7D: p<0.0001; iDC + mock lysate vs iDC +vOka: p<0.0001<br>CD86: iDC + mock lysate vs iDC +v7D: p<0.0001; iDC + mock lysate vs iDC +vOka: p<0.0001
4h	n=3 per group	One-way ANOVA with Tukey's post-hoc test	CD4+ T cell: iDC + mock lysate vs iDC +v7D: p=0.0185; iDC + mock lysate vs iDC +vOka: p=0.0077<br>CD8+ T cell: iDC + mock lysate vs iDC +v7D: p<0.0001; iDC + mock lysate vs iDC +vOka: p<0.0001
4i	n=3 per group	One-way ANOVA with Tukey's post-hoc test	CD4+ T cell: iDC + mock lysate vs iDC +v7D: p=0.0261; iDC + mock lysate vs iDC +vOka: p=0.0117<br>CD8+ T cell: iDC + mock lysate vs iDC +v7D: p=0.0144; iDC + mock lysate vs iDC +vOka: p=0.0109
5c	n=5 per group	Two-way ANOVA with Tukey's post-hoc test	Mouse IgG1, week 9, Mock vs v7D: p=0.001; Mock vs vOka: p=0.0002<br>Mouse IgG2a, week 9, Mock vs v7D: p=0.0003; Mock vs vOka: p=0.0018<br>Mouse IgG2b, week 9, Mock vs v7D: p=0.0041; Mock vs vOka: p<0.0001<br>Mouse IgG2c, week 9, Mock vs v7D: p=0.5964; Mock vs vOka: p=0.0553

Figure	Sample size (n)	Statistical Test	Values
5d	n=5 per group	Two-way ANOVA with Tukey's post-hoc test	Rat IgG1, week 9, Mock vs v7D: p=0.0046; Mock vs vOka: p=0.0241<br>Rat IgG2a, week 9, Mock vs v7D: p=0.0016; Mock vs vOka: p=0.0002<br>Rat IgG2b, week 9, Mock vs v7D: p=0.0115; Mock vs vOka: p=0.0017<br>Rat IgG2c, week 9, Mock vs v7D: p=0.0054; Mock vs vOka: p=0.0307
S6a	n=3 per group	One-way ANOVA with Tukey's post-hoc test	TNF-α: iDC + mock lysate vs iDC +v7D: p<0.0001; iDC + mock lysate vs iDC +vOka: p<0.0001<br>IFNγ: iDC + mock lysate vs iDC +v7D: p<0.0001; iDC + mock lysate vs iDC +vOka: p<0.0001<br>IL-2: iDC + mock lysate vs iDC +v7D: p<0.0001; iDC + mock lysate vs iDC +vOka: p<0.0001<br>IL-4: iDC + mock lysate vs iDC +v7D: p=0.0003; iDC + mock lysate vs iDC +vOka: p=0.0014<br>IL-6: iDC + mock lysate vs iDC +v7D: p<0.0001; iDC + mock lysate vs iDC +vOka: p<0.0001<br>IL-8: iDC + mock lysate vs iDC +v7D: p=0.0139; iDC + mock lysate vs iDC +vOka: p=0.0296<br>IL-1ra: iDC + mock lysate vs iDC +v7D: p<0.0001; iDC + mock lysate vs iDC +vOka: p<0.0001<br>IP-10: iDC + mock lysate vs iDC +v7D: p=0.004; iDC + mock lysate vs iDC +vOka: p=0.0052<br>GM-CSF: iDC + mock lysate vs iDC +v7D: p<0.0001; iDC + mock lysate vs iDC +vOka: p<0.0001<br>VEGF: iDC + mock lysate vs iDC +v7D: p=0.0038; iDC + mock lysate vs iDC +vOka: p=0.016

Figure	Sample size (n)	Statistical Test	Values
S6b	n=3 per group	One-way ANOVA with Tukey's post-hoc test	TNF-α: iDC + mock lysate vs iDC +v7D: p<0.0001; iDC + mock lysate vs iDC +vOka: p<0.0001<br>IFNγ: iDC + mock lysate vs iDC +v7D: p<0.0001; iDC + mock lysate vs iDC +vOka: p<0.0001<br>IL-2: iDC + mock lysate vs iDC +v7D: p<0.0001; iDC + mock lysate vs iDC +vOka: p<0.0001<br>IL-1ra: iDC + mock lysate vs iDC +v7D: p<0.0001; iDC + mock lysate vs iDC +vOka: p<0.0001<br>IP-10: iDC + mock lysate vs iDC +v7D: p=0.015; iDC + mock lysate vs iDC +vOka: p=0.0348<br>GM-CSF: iDC + mock lysate vs iDC +v7D: p=0.0003; iDC + mock lysate vs iDC +vOka: p=0.0009
5e, S7	n=3 per group	Two-way ANOVA with Tukey's post-hoc test	TNF-α mock: naïve vs v7D: p=0.0621<br>naïve vs vOka: p=0.0323<br>TNF-α VZV: naïve vs v7D: p=0.0001<br>naïve vs vOka: p=0.001<br>IFN-γ VZV: naïve vs v7D: p<0.0001<br>naïve vs vOka: p=0.0001<br>IL-1β mock: naïve vs v7D: p=0.1001<br>naïve vs vOka: p=0.0057<br>IL-1β VZV: naïve vs v7D: p<0.0001<br>naïve vs vOka: p<0.0001<br>IL-2 VZV: naïve vs v7D: p<0.0001<br>naïve vs vOka: p<0.0001<br>IL-3 VZV: naïve vs v7D: p=0.0002<br>naïve vs vOka: p=0.0002<br>IL-4 mock: naïve vs v7D: p=0.0206<br>naïve vs vOka: p=0.0240<br>IL-4 VZV: naïve vs v7D: p=0.0125<br>naïve vs vOka: p=0.0025<br>IL-5 VZV: naïve vs v7D: p<0.0001<br>naïve vs vOka: p<0.0001<br>IL-6 mock: naïve vs v7D: p=0.0006<br>naïve vs vOka: p<0.0001<br>IL-6 VZV: naïve vs v7D: p<0.0001<br>naïve vs vOka: p<0.0001

Figure	Sample size (n)	Statistical Test	Values
5e, S7	n=3 per group	Two-way ANOVA with Tukey's post-hoc test	IL-9 mock: naïve vs v7D: p=0.0028<br>naïve vs vOka: p=0.0344<br>IL-9 VZV: naïve vs v7D: p=0.0072<br>naïve vs vOka: p=0.0022<br>IL-10 mock: naïve vs v7D: p=0.1711<br>naïve vs vOka: p=0.0202<br>IL-12p70 mock: naïve vs v7D: p=0.0006<br>naïve vs vOka: p=0.0153<br>IL-12p70 VZV: naïve vs v7D: p=0.0405<br>naïve vs vOka: p=0.0102<br>IL-15 VZV: naïve vs v7D: p=0.0003<br>naïve vs vOka: p=0.0002<br>IP-10 VZV: naïve vs v7D: p=0.0009<br>naïve vs vOka: p=0.0074<br>GM-CSF mock: naïve vs v7D: p=0.0179<br>naïve vs vOka: p=0.0343<br>GM-CSF VZV: naïve vs v7D: p=0.2067<br>naïve vs vOka: p=0.0004<br>M-CSF mock: naïve vs v7D: p<0.0001<br>naïve vs vOka: p<0.0001<br>M-CSF VZV: naïve vs v7D: p=0.0029<br>naïve vs vOka: p=0.0170<br>MIP-1α mock: naïve vs v7D: p<0.0001<br>naïve vs vOka: p<0.0001<br>MIP-1α VZV: naïve vs v7D: p=0.0331<br>naïve vs vOka: p=0.0417<br>MIP-1β mock: naïve vs v7D: p<0.0001<br>naïve vs vOka: p<0.0001<br>MIP-1β VZV: naïve vs v7D: p<0.0001<br>naïve vs vOka: p<0.0001<br>MIP-2 mock: naïve vs v7D: p<0.0001<br>naïve vs vOka: p<0.0001<br>LIF VZV: naïve vs v7D: p=0.0001<br>naïve vs vOka: p<0.0001<br>LIX VZV: naïve vs v7D: p<0.0001<br>naïve vs vOka: p<0.0001<br>RANTES VZV: naïve vs v7D: p<0.0001<br>naïve vs vOka: p<0.0001
5f, S8	n=3 per group	Two-way ANOVA with Tukey's post-hoc test	TNF-α VZV: naïve vs v7D: p<0.0001<br>naïve vs vOka: p<0.0001<br>IFN-γ VZV: naïve vs v7D: p<0.0001<br>naïve vs vOka: p<0.0001

Figure	Sample size (n)	Statistical Test	Values
5f, S8	n=3 per group	Two-way ANOVA with Tukey's post-hoc test	IL-1α VZV: naïve vs v7D: $p<0.0001$<br>naïve vs vOka: $p<0.0001$<br>IL-2 VZV: naïve vs v7D: $p=0.0070$<br>naïve vs vOka: $p=0.0115$<br>IL-5 VZV: naïve vs v7D: $p=0.0141$<br>naïve vs vOka: $p=0.0340$<br>IL-6 VZV: naïve vs v7D: $p<0.0001$<br>naïve vs vOka: $p<0.0001$<br>IL-10 VZV: naïve vs v7D: $p<0.0001$<br>naïve vs vOka: $p<0.0001$<br>IL-13 VZV: naïve vs v7D: $p<0.0001$<br>naïve vs vOka: $p<0.0001$<br>IL-17A VZV: naïve vs v7D: $p<0.0001$<br>naïve vs vOka: $p<0.0001$<br>IL-18 mock: naïve vs v7D: $p=0.0497$<br>naïve vs vOka: $p=0.2173$<br>IL-18 VZV: naïve vs v7D: $p<0.0001$<br>naïve vs vOka: $p<0.0001$<br>GM-CSF mock: naïve vs v7D: $p=0.0007$<br>naïve vs vOka: $p=0.0042$<br>GM-CSF VZV: naïve vs v7D: $p<0.0001$<br>naïve vs vOka: $p<0.0001$<br>LIX mock: naïve vs v7D: $p<0.0001$<br>naïve vs vOka: $p<0.0001$<br>LIX VZV: naïve vs v7D: $p<0.0001$<br>naïve vs vOka: $p<0.0001$<br>MCP-1 mock: naïve vs v7D: $p=0.0147$<br>naïve vs vOka: $p=0.0081$<br>MCP-1 VZV: naïve vs v7D: $p<0.0001$<br>naïve vs vOka: $p<0.0001$<br>MIP-1α VZV: naïve vs v7D: $p<0.0001$<br>naïve vs vOka: $p<0.0001$<br>MIP-2 mock: naïve vs v7D: $p<0.0001$<br>naïve vs vOka: $p<0.0001$<br>MIP-2 VZV: naïve vs v7D: $p=0.5930$<br>naïve vs vOka: $p=0.0179$<br>KC VZV: naïve vs v7D: $p<0.0001$<br>naïve vs vOka: $p<0.0001$<br>RANTES mock: naïve vs v7D: $p=0.0213$<br>naïve vs vOka: $p=0.0046$<br>RANTES VZV: naïve vs v7D: $p<0.0001$<br>naïve vs vOka: $p<0.0001$

Figure	Sample size (n)	Statistical Test	Values
S9	n=3 per group	Two-way ANOVA with Tukey's post-hoc test	TNF-α mock: naïve vs v7D: p<0.0001<br>naïve vs vOka: p<0.0001<br>TNF-α VZV: naïve vs v7D: p<0.0001<br>naïve vs vOka: p=0.0006<br>IFN-γ mock: naïve vs v7D: p<0.0001<br>naïve vs vOka: p<0.0001<br>IFN-γ VZV: naïve vs v7D: p<0.0001<br>naïve vs vOka: p<0.0001<br>IL-1α mock: naïve vs v7D: p<0.0001<br>naïve vs vOka: p<0.0001<br>IL-1α VZV: naïve vs v7D: p<0.0001<br>naïve vs vOka: p<0.0001<br>IL-1β mock: naïve vs v7D: p=0.0080<br>naïve vs vOka: p<0.0001<br>IL-1β VZV: naïve vs v7D: p<0.0001<br>naïve vs vOka: p<0.0001<br>IL-2 mock: naïve vs v7D: p<0.0001<br>naïve vs vOka: p<0.0001<br>IL-2 VZV: naïve vs v7D: p<0.0001<br>naïve vs vOka: p<0.0001<br>IL-3 mock: naïve vs v7D: p<0.0001<br>naïve vs vOka: p<0.0001<br>IL-3 VZV: naïve vs v7D: p<0.0001<br>naïve vs vOka: p<0.0001<br>IL-4 mock: naïve vs v7D: p<0.0001<br>naïve vs vOka: p<0.0001<br>IL-4 VZV: naïve vs v7D: p<0.0001<br>naïve vs vOka: p<0.0001<br>IL-5 mock: naïve vs v7D: p<0.0001<br>naïve vs vOka: p<0.0001<br>IL-5 VZV: naïve vs v7D: p<0.0001<br>naïve vs vOka: p<0.0001<br>IL-6 mock: naïve vs v7D: p<0.0001<br>naïve vs vOka: p<0.0001<br>IL-6 VZV: naïve vs v7D: p<0.0001<br>naïve vs vOka: p<0.0001<br>IL-10 mock: naïve vs v7D: p<0.0001<br>naïve vs vOka: p<0.0001<br>IL-10 VZV: naïve vs v7D: p<0.0001<br>naïve vs vOka: p<0.0001<br>IL-12p70 mock: naïve vs v7D: p=0.0032<br>naïve vs vOka: p=0.0045

Figure	Sample size (n)	Statistical Test	Values
S9	n=3 per group	Two-way ANOVA with Tukey's post-hoc test	IL-17 mock: naïve vs v7D: p=0.0098<br>naïve vs vOka: p=0.0734<br>IL-17 VZV: naïve vs v7D: p<0.0001<br>naïve vs vOka: p<0.0001<br>IP-10 mock: naïve vs v7D: p<0.0001<br>naïve vs vOka: p<0.0001<br>IP-10 VZV: naïve vs v7D: p=0.0016<br>naïve vs vOka: p=0.0014<br>G-CSF VZV: naïve vs v7D: p<0.0001<br>naïve vs vOka: p<0.0001<br>LIF mock: naïve vs v7D: p=0.0077<br>naïve vs vOka: p=0.0061<br>LIF VZV: naïve vs v7D: p=0.0001<br>naïve vs vOka: p=0.0016<br>MIP-2 mock: naïve vs v7D: p<0.0001<br>naïve vs vOka: p<0.0001<br>MIP-2 VZV: naïve vs v7D: p<0.0001<br>naïve vs vOka: p<0.0001<br>MIG mock: naïve vs v7D: p=0.0016<br>naïve vs vOka: p<0.0001<br>MIG VZV: naïve vs v7D: p=0.0069<br>naïve vs vOka: p=0.0069<br>MIP-1α mock: naïve vs v7D: p=0.0009<br>naïve vs vOka: p=0.0010<br>MIP-1β mock: naïve vs v7D: p<0.0001<br>naïve vs vOka: p<0.0001<br>MIP-1β VZV: naïve vs v7D: p=0.0095<br>naïve vs vOka: p=0.3981<br>VEGF VZV: naïve vs v7D: p<0.0001<br>naïve vs vOka: p<0.0001
S10	n=3 per group	Two-way ANOVA with Tukey's post-hoc test	TNF-α VZV: naïve vs v7D: p=0.0190<br>naïve vs vOka: p=0.0143<br>IFN-γ mock: naïve vs v7D: p=0.0007<br>naïve vs vOka: p=0.0033<br>IFN-γ VZV: naïve vs v7D: p<0.0001<br>naïve vs vOka: p<0.0001<br>IL-1α VZV: naïve vs v7D: p=0.0001<br>naïve vs vOka: p=0.0019<br>IL-1β VZV: naïve vs v7D: p<0.0001<br>IL-2 VZV: naïve vs v7D: p<0.0001<br>naïve vs vOka: p<0.0001<br>IL-4 VZV: naïve vs v7D: p<0.0001

Figure	Sample size (n)	Statistical Test	Values
S10	n=3 per group	Two-way ANOVA with Tukey's post-hoc test	IL-4 VZV: naïve vs vOka: p<0.0001<br>IL-6 VZV: naïve vs v7D: p=0.0107<br>naïve vs vOka: p=0.0036<br>IL-9 mock: naïve vs v7D: p=0.0013<br>naïve vs vOka: p=0.0013<br>IL-9 VZV: naïve vs v7D: p<0.0001<br>naïve vs vOka: p<0.0001<br>IL-12p70 mock: naïve vs v7D: p=0.0029<br>naïve vs vOka: p=0.0100<br>IL-12p70 VZV: naïve vs v7D: p<0.0001<br>naïve vs vOka: p<0.0001<br>IP-10 VZV: naïve vs v7D: p<0.0001<br>naïve vs vOka: p<0.0001<br>G-CSF VZV: naïve vs v7D: p<0.0001<br>naïve vs vOka: p=0.0007<br>MIP-1α VZV: naïve vs v7D: p<0.0001<br>naïve vs vOka: p<0.0001<br>KC VZV: naïve vs v7D: p=0.0001<br>naïve vs vOka: p<0.0001<br>RANTES VZV: naïve vs v7D: p<0.0001<br>naïve vs vOka: p<0.0001
S11	n=3 per group	Two-way ANOVA with Tukey's post-hoc test	TNF-α VZV: naïve vs v7D: p<0.0001<br>naïve vs vOka: p<0.0001<br>IFN-γ VZV: naïve vs v7D: p<0.0001<br>naïve vs vOka: p<0.0001<br>IL-1α VZV: naïve vs v7D: p<0.0001<br>naïve vs vOka: p<0.0001<br>IL-2 VZV: naïve vs v7D: p<0.0001<br>naïve vs vOka: p<0.0001<br>IL-10 mock: naïve vs v7D: p=0.0107<br>naïve vs vOka: p=0.0004<br>IL-10 VZV: naïve vs v7D: p<0.0001<br>naïve vs vOka: p<0.0001<br>IL-17A VZV: naïve vs v7D: p=0.0011<br>naïve vs vOka: p=0.0063<br>IL-18 VZV: naïve vs v7D: p<0.0001<br>naïve vs vOka: p<0.0001<br>GM-CSF VZV: naïve vs v7D: p<0.0001<br>naïve vs vOka: p<0.0001<br>LIX VZV: naïve vs v7D: p=0.0012<br>naïve vs vOka: p=0.0002<br>MCP-1 VZV: naïve vs v7D: p<0.0001

Figure	Sample size (n)	Statistical Test	Values
S11	n=3 per group	Two-way ANOVA with Tukey's post-hoc test	naïve vs vOka: p<0.0001<br>MIP-1α VZV: naïve vs v7D: p<0.0001<br>naïve vs vOka: p<0.0001<br>MIP-2 mock: naïve vs v7D: p<0.0001<br>naïve vs vOka: p<0.0001<br>KC VZV: naïve vs v7D: p=0.0001<br>naïve vs vOka: p<0.0001<br>RANTES VZV: naïve vs v7D: p<0.0001<br>naïve vs vOka: p<0.0001
S12	n=3 per group	Two-way ANOVA with Tukey's post-hoc test	TNF-α VZV: naïve vs v7D: p=0.0144<br>naïve vs vOka: p=0.0103<br>IFN-γ VZV: naïve vs v7D: p=0.0044<br>naïve vs vOka: p=0.0020<br>IL-1α mock: naïve vs v7D: p<0.0001<br>naïve vs vOka: p<0.0001<br>IL-1α VZV: naïve vs v7D: p<0.0001<br>naïve vs vOka: p<0.0001<br>IL-1β VZV: naïve vs v7D: p<0.0001<br>naïve vs vOka: p<0.0001<br>IL-2 VZV: naïve vs v7D: p=0.0045<br>naïve vs vOka: p=0.0066<br>IL-4 VZV: naïve vs v7D: p=0.0004<br>naïve vs vOka: p=0.0010<br>IL-6 VZV: naïve vs v7D: p<0.0001<br>naïve vs vOka: p<0.0001<br>IL-10 mock: naïve vs v7D: p=0.0005<br>naïve vs vOka: p=0.0016<br>IL-10 VZV: naïve vs v7D: p<0.0001<br>naïve vs vOka: p<0.0001<br>IP-10 VZV: naïve vs v7D: p<0.0001<br>naïve vs vOka: p<0.0001<br>Leptin VZV: naïve vs v7D: p<0.0001<br>naïve vs vOka: p<0.0001<br>LIX mock: naïve vs v7D: p=0.0004<br>naïve vs vOka: p=0.0055<br>LIX VZV: naïve vs v7D: p<0.0001<br>naïve vs vOka: p<0.0001<br>MCP-1 mock: naïve vs v7D: p=0.0048<br>naïve vs vOka: p=0.0027<br>MCP-1 VZV: naïve vs v7D: p<0.0001<br>naïve vs vOka: p<0.0001<br>MIP-2 mock: naïve vs v7D: p=0.0050

Figure	Sample size (n)	Statistical Test	Values
S12	n=3 per group	Two-way ANOVA with Tukey's post-hoc test	MIP-2 mock: naïve vs vOka: p=0.0051<br>MIP-2 VZV: naïve vs v7D: p<0.0001<br>naïve vs vOka: p<0.0001<br>KC VZV: naïve vs v7D: p<0.0001<br>naïve vs vOka: p<0.0001<br>RANTES mock: naïve vs v7D: p=0.0099<br>naïve vs vOka: p=0.001<br>RANTES VZV: naïve vs v7D: p<0.0001<br>naïve vs vOka: p<0.0001<br>VEGF mock: naïve vs v7D: p<0.0001<br>naïve vs vOka: p<0.0001<br>VEGF VZV: naïve vs v7D: p<0.0001<br>naïve vs vOka: p<0.0001
S13a	n=3 per group	Two-way ANOVA with Tukey's post-hoc test	TNF-α VZV: naïve vs v7D: p<0.0001<br>naïve vs vOka: p<0.0001
S13b	n=3 per group	Two-way ANOVA with Tukey's post-hoc test	TNF-α VZV: naïve vs v7D: p<0.0001<br>naïve vs vOka: p<0.0001

Supplementary Table 4. Primers for VZV-7D BAC construction: (1) Replacement of ORF7 to galK.

Name	Primers
ORF7-galK-F	CGTTGGAAAGCCAGTCAATCCCTGTTGACAATTAATCATCGGC
ORF7-galK-R	TTTAGTTTTTATTTCAATATTCAGCACTGTCCTGCTCCTT
ORF7-up350-galK-F	CCGGGATCGTACATCTCCAG
ORF7-up350-galK-R	GCCGATGATTAATTGTCAACAGGGATTGACTGGCTTTCCAACG
ORF7-down350-galK-F	AAGGAGCAGGACAGTGCTGAATATTGAAATAAAAACTAAA
ORF7-down350-galK-R	ATGACATACGTATAAAAACG

Supplementary Table 5. Primers for VZV-7D BAC construction: (2) Replacement of galK to Stop-ORF7.

Name	Primers
ORF7 stop-F	CGTTGGAAAGCCAGTCAATCATGTAGCTAGCTAGTGCCAGCTTATGTGGATA
ORF7 stop-R	TTTAGTTTTTATTTCAATATTTATACAAGCATAACATGGG
ORF7-up350-stop-F	CCGGGATCGTACATCTCCAG
ORF7-up350-stop-R	CTGGCACTAGCTAGCTACATGATTGACTGGCTTTCCAACG
ORF7-down350-stop-F	CCCATGTTATGCTTGTATAAATATTGAAATAAAAACTAAA
ORF7-down350-stop-R	ATGACATACGTATAAAAACG

Supplementary Table 6. Primers for RT-PCR.

Name	Primers
Human GAPDH mRNA-F	ACCCACTCCTCCACCTTTGAC
Human GAPDH mRNA-R	CTGTTGCTGTAGCCAAATTCGT
Guinea pig GAPDH mRNA-F	AATGGGAAGCTCACAGGTATGG
Guinea pig GAPDH mRNA-R	ATGTCATCGTATTTGGCCGGT
Cotton rat GAPDH mRNA-F	AAGAGTTATCATCTCCGCCC
Cotton rat GAPDH mRNA-R	AATGCCAAAGTTGTCGTGGAT
VZV-ORF31 mRNA-F	GCCGTGGGATTATTGGTTT
VZV-ORF31 mRNA-R	AGTAGCGTTGGGTTTCTCG
VZV-ORF62 mRNA-F	ATGTGGTTTCCAAGGCCAAGAG
VZV-ORF62 mRNA-R	TCCGTCAAGTGGCATCGTTATT

Supplementary Table 7. Primers for qPCR.

Name	Primers
Human GAPDH qPCR-F	TATTGGGCGCCTGGTC
Human GAPDH qPCR-R	GACGGTGCCATGGAATT
Human GAPDH qPCR-probe	**FAM**-TTAACTCTGGTAAAGTGGATATTGTTGCC-**TAMRA**
Guinea pig GAPDH qPCR-F	AATGGGAAGCTCACAGGTATGG
Guinea pig GAPDH qPCR-R	ATGTCATCGTATTTGGCCGGT
Guinea pig GAPDH qPCR-probe	**FAM**-TCCAGGCGGCAGGTCAGATCCACA-**TAMR**A
Cotton rat GAPDH qPCR-F	AAGAGTTATCATCTCCGCCC
Cotton rat GAPDH qPCR-R	AATGCCAAAGTTGTCGTGGAT
Cotton rat GAPDH qPCR-probe	**FAM**-AACAATGCTTCCTGCACCACCAACTGC-**TAMRA**
VZV-ORF31 qPCR-F	GATGGTGCATACAGAGAACATTCC
VZV-ORF31 qPCR-R	CCGTTAAATGAGGCGTGACTAA
VZV-ORF31 qPCR-probe	**FAM**-TCCGCGCTGCAGGTTCCAGTAAT-**TAMRA**
VZV-ORF62 qPCR-F	TCTTGTCGAGGAGGCTTCTG
VZV-ORF62 qPCR-R	TGTGTGTCCACCGGATGAT
VZV-ORF62 qPCR-probe	**FAM**-TCTCGACTGGCTGGGACTTGCG-**TAMRA**
