# Supplementary material for: Development of a skin- and neuro-attenuated live vaccine for varicella
Source: Nat Commun. 2022 Feb 11;13:824. doi: 10.1038/s41467-022-28329-1 (PMC8837607; doi:10.1038/s41467-022-28329-1)
Supplement: Supplementary file 3 — Reporting Summary [file 41467_2022_28329_MOESM3_ESM.pdf]

nature research

Corresponding author(s): Ningshao Xia
Last updated by author(s): Jan 7, 2022

Reporting Summary

Nature Research wishes to improve the reproducibility of the work that we publish. This form provides structure for consistency and transparency in reporting. For further information on Nature Research policies, see Authors & Referees and the Editorial Policy Checklist .

Statistics

For all statistical analyses, confirm that the following items are present in the figure legend, table legend, main text, or Methods section.

n/a	Confirmed	
☐	☒	The exact sample size (*n*) for each experimental group/condition, given as a discrete number and unit of measurement
☐	☒	A statement on whether measurements were taken from distinct samples or whether the same sample was measured repeatedly
☐	☒	The statistical test(s) used AND whether they are one- or two-sided<br>*Only common tests should be described solely by name; describe more complex techniques in the Methods section.*
☐	☒	A description of all covariates tested
☐	☒	A description of any assumptions or corrections, such as tests of normality and adjustment for multiple comparisons
☐	☒	A full description of the statistical parameters including central tendency (e.g. means) or other basic estimates (e.g. regression coefficient) AND variation (e.g. standard deviation) or associated estimates of uncertainty (e.g. confidence intervals)
☐	☒	For null hypothesis testing, the test statistic (e.g. *F*, *t*, *r*) with confidence intervals, effect sizes, degrees of freedom and *P* value noted<br>*Give P values as exact values whenever suitable.*
☒	☐	For Bayesian analysis, information on the choice of priors and Markov chain Monte Carlo settings
☒	☐	For hierarchical and complex designs, identification of the appropriate level for tests and full reporting of outcomes
☒	☐	Estimates of effect sizes (e.g. Cohen's *d*, Pearson's *r*), indicating how they were calculated

Our web collection on statistics for biologists contains articles on many of the points above.

Software and code

Policy information about availability of computer code

Data collection	Confocal images were acquired using a Zeiss LSM 780 confocal microscope with the ZEN software (version 2.3).<br>Flow cytometry data were acquired using a FACSAria flow cytometer with the FACSDiva software (version 6.1.3).<br>Q-PCR was performed using a Bio-rad CFX96 Real-time System with the CFX Maestro software (version 4.1.2433.1219).
Data analysis	Statistical analysis was performed with the GraphPad Prism software (version 7).<br>Flow cytometry data were analyzed with the FACSDiva software (version 6.1.3) and FlowJo (version 10).

For manuscripts utilizing custom algorithms or software that are central to the research but not yet described in published literature, software must be made available to editors/reviewers. We strongly encourage code deposition in a community repository (e.g. GitHub). See the Nature Research guidelines for submitting code & software for further information.

Data

Policy information about availability of data

All manuscripts must include a data availability statement. This statement should provide the following information, where applicable:
- Accession codes, unique identifiers, or web links for publicly available datasets
- A list of figures that have associated raw data
- A description of any restrictions on data availability

All data associated with this study are present in the paper or the Supplementary Information. Source data are provided with this paper.

Field-specific reporting

Please select the one below that is the best fit for your research. If you are not sure, read the appropriate sections before making your selection.

Life sciences study design

All studies must disclose on these points even when the disclosure is negative.

Sample size	No statistical tests were performed to pre-determine sample size. Sample sizes for evaluation of virus infection in the SCID-hu mouse models as well as the guinea pig and cotton rat models were selected based on experiences published in the literatures (Zerboni et al., 2005, PNAS, PMID: 15851670; Moffat et al., 1998, J. Virol., PMID: 9444989; Cohen et al., 2004, J. Virol., PMID: 15479825; Lowry et al., 1993, J. Virol., PMID: 8380293). Sample sizes for evaluation of toxicity and/or immunogenicity in different animal models were selected based on lab experience to ensure the reproducibility and to perform statistical analysis. For in vitro experiments, at least 3 biological replicates per condition was used to perform statistical analysis.
Data exclusions	No data points were excluded from data sets.
Replication	Experiments were carried out at least two times independently and were successfully reproducible.
Randomization	The allocation into experimental groups was random in all animal experiments.
Blinding	For vaccination experiments in small animals, the investigators were blinded to the type of the vaccine used. For toxicity evaluation in nonhuman primates, histopathological analysis was performed by veterinary pathologists who were blinded to treatment groups. All experimental procedures and data collection or analysis were done by two independent investigators.

Reporting for specific materials, systems and methods

We require information from authors about some types of materials, experimental systems and methods used in many studies. Here, indicate whether each material, system or method listed is relevant to your study. If you are not sure if a list item applies to your research, read the appropriate section before selecting a response.

Materials & experimental systems

n/a	Involved in the study	
☐	☒	Antibodies
☐	☒	Eukaryotic cell lines
☒	☐	Palaeontology
☐	☒	Animals and other organisms
☐	☒	Human research participants
☒	☐	Clinical data

Methods

n/a	Involved in the study	
☒	☐	ChIP-seq
☐	☒	Flow cytometry
☒	☐	MRI-based neuroimaging

Antibodies

Antibodies used

Primary antibodies:

(1) Commercial antibodies: Anti-CD4 (561843, dilution 1:20) and anti-CD8 (561949, dilution 1:20) antibodies were purchased from BD Biosciences for Flow cytometry. Anti-CD14 (MHCD1401, dilution 1:1000), anti-CD40 (CD4004, dilution 1:20), anti-CD80 (MA1-19590, dilution 1:20), anti-CD83 (MHCD8304, dilution 1:20) and anti-CD86 (MA1-10296, dilution 1:20) antibodies were purchased from Thermo Fisher Scientific for Flow cytometry. Anti-GAPDH antibody (60004-1-Ig, dilution 1:1000) was purchased from Proteintech for Western Blot analysis. Anti-VZV glycoprotein I (ab52552, dilution 1:1000) was purchased from Abcam for Western Blot analysis. Anti-cleaved PARP (9541S, dilution 1:500) and anti-cleaved caspase-3 (9664S, dilution 1:500) were purchased from Cell Signaling Technology for Western Blot analysis.

(2) Most antibodies against VZV viral proteins were prepared from our own lab, including anti-pORF7 (clone 8H3,dilution 1:1000 for Western Blot; a rabbit polyclonal antibody 7M, dilution 1:500 for IFA), anti-pORF9 (clone 8H6, dilution 1:1000 for Western Blot), anti-gE (clone 4A2, dilution 1:1000 for Western Blot; dilution 1:200 for Flow cytometry; dilution 1:2000 for IHC), anti-gB (clone 10E10, dilution 1:1000 for Western Blot), anti-gH (clone 10F5, dilution 1:1000 for Western Blot), anti-gN (clone 12E10, dilution 1:1000 for Western Blot), anti-pORF23 (clone 9A1, dilution 1:1000 for Western Blot), anti-pORF40 (clone 10A2, dilution 1:1000 for Western Blot), anti-pORF47 (clone 11H4, dilution 1:1000 for Western Blot), anti-pORF62 (clone 1B7, dilution 1:1000 for Western Blot; dilution 1:2000 for IFA; dilution 1:5000 for IHC) and anti-pORF63 (clone 1B1, dilution 1:1000 for Western Blot) antibodies.

Secondary antibodies: HRP-conjugated goat anti-mouse (31430, dilution 1:5000), anti-rabbit (31460, dilution 1:5000) and anti-rat (31470, dilution 1:5000) total IgG, HRP-conjugated goat-anti mouse IgG2c (PA1-29288, dilution 1:10000), and HRP-conjugated goat anti-rat IgG1 (PA1-84708, dilution 1:10000), IgG2a (PA1-84709, dilution 1:10000), IgG2b (PA1-84710, dilution 1:10000) and IgG2c (PA1-84711, dilution 1:10000) secondary antibodies were all purchased from Thermo Fisher Scientific for ELISA. HRP-conjugated goat anti-mouse IgG1 (ab98693, dilution 1:10000), IgG2a (ab98698, dilution 1:10000) and IgG2b (ab97250, dilution 1:10000), and HRP-conjugated goat anti-guinea pig (ab6908, dilution 1:100000) and goat anti-monkey (ab112767, dilution 1:5000) total IgG secondary antibodies were purchased from Abcam for ELISA. Goat-anti-rabbit-FITC (F9887, dilution 1:500) and goat-anti-mouse-TRITC (T5393, dilution 1:200)-labeled secondary antibodies were purchased from Sigma for IFA.

Validation	All antibodies were validated by the manufacturers and by our own and colleagues' labs.<br>(1) For antibodies that made in our own lab, including mouse monoclonal antibodies against pORF7 (8H3), pORF9 (8H6), pORF23 (9A1), pORF40 (10A2), pORF47 (11H4), pORF62 (1B7), pORF63 (1B1), gE (4A2), gB (10E10), gH(10F5), gN (12E10) and a rabbit polyclonal antibody against pORF7 (7M), validations have been done by Western Blot, IFA, IHC and/or Flow cytometry, which are provided in the manuscript.<br>(2) For antibodies that are commercially available, validation data can be found in each of the company's website. We followed the manufactures' instruction to use these commercial antibodies, and all antibodies worked well in this study.

Eukaryotic cell lines

Policy information about cell lines

Cell line source(s)	MRC-5 cells (CCL-171) and SH-SY5Y cells (CRL-2266) were purchased from ATCC.<br>Human dermal fibroblasts (2310) and human epidermal keratinocytes (2100) were purchased from ScienCell Research Laboratories.
Authentication	MRC-5 cells and SH-SY5Y cells were authenticated by STR testing, conducted by ATCC.<br>Human dermal fibroblasts and epidermal keratinocytes were authenticated by morphology and immunophenotyping, conducted by ScienCell Research Laboratories.
Mycoplasma contamination	All cell lines were tested to be negative for mycoplasma contamination.
Commonly misidentified lines (See ICLAC register)	No commonly misidentified cell lines were used in this study.

Animals and other organisms

Policy information about studies involving animals; ARRIVE guidelines recommended for reporting animal research

Laboratory animals	For the SCID-hu mouse model, 4- to 6-week-old male CB-17 SCID mice were used.<br>For vaccination experiments, 4- to 6-week-old female BALB/c mice, 6- to 8-week-old female SD rats, 12- to 14-week-old female New Zealand White rabbits, and 10- to 12-week-old female Hartley guinea pigs were used.<br>For preclinical safety evaluations, 2.5- to 3-year-old rhesus macaques and 2- to 5-year-old cynomolgus macaques (male:female ratio 1:1) were used.
Wild animals	The study did not involve wild type animals.
Field-collected samples	The study did not involve samples collected from the field.
Ethics oversight	For vaccine safety evaluation in SCID-hu mice, the use of the human tissues was approved by the Research Ethics Committee of Xiamen University (Approval NO. SPH-XMU2016006). Animal experiments were carried out under specific-pathogen-free (SPF) conditions and in strict accordance with the approved animal use protocols of Xiamen University Laboratory Animal Center (Approval NO. XMULAC20160050).<br>For vaccination experiments, all animals were maintained and handled in accordance with standard use protocols and animal welfare regulations of Xiamen University Laboratory Animal Center (Approval NO. XMULAC20160050).<br>For preclinical safety evaluations, all animals were maintained at the animal facility of Joinn Laboratories (China) Co. Ltd (Beijing, China). The non-human primate study protocol and all the experimental procedures were reviewed and approved by the local Institutional Animal Care and Use Committee (NO. ACU16-040 and ACU15-999).

Note that full information on the approval of the study protocol must also be provided in the manuscript.

Human research participants

Policy information about studies involving human research participants

Population characteristics	The study used PBMCs from 6 healthy donors of age 20–50 years.
Recruitment	PBMCs were obtained commercially from Hemacare (Van Nuys, CA, USA, PB009C). The healthy donors providing PBMCs were screened for infectious diseases and found negative for hepatitis B virus (HBV) surface antigen, HBV core antibody, hepatitis C virus (HCV) antibody, human immunodeficiency virus -1/2 (HIV-1/2) antibody, HIV-1/HCV/HBV nucleic acid testing, and syphilis. Informed consent was obtained by the source company from all adult subjects providing PBMCs, and samples were de-identified by the company prior to our receipt.
Ethics oversight	The use of human PBMCs in this study was approved by the Research Ethics Committee of Xiamen University (Approval NO. SPH-XMU2016006).

Note that full information on the approval of the study protocol must also be provided in the manuscript.

Flow Cytometry

Plots

Confirm that:

- [x] The axis labels state the marker and fluorochrome used (e.g. CD4-FITC).
- [x] The axis scales are clearly visible. Include numbers along axes only for bottom left plot of group (a 'group' is an analysis of identical markers).
- [x] All plots are contour plots with outliers or pseudocolor plots.
- [x] A numerical value for number of cells or percentage (with statistics) is provided.

Methodology

Sample preparation	Mock-infected and VZV-infected PBMCs and DCs were washed and then incubated with antibodies against cell surface antigens.
Instrument	BD FACSAria flow cytometer was used for flow cytometry data collection.
Software	BD FACSDiva software (version 6.1.3) and FlowJo (version 10).
Cell population abundance	Acquisition was performed on >20,000 events in the FSC-SSC gate per sample.
Gating strategy	Dead cells were gated out using forward scatter-side scatter (FSC-SSC) profiles. The gates positive for indicated cell surface antigens were obtained by comparing to the uninfected and isotype controls.

- [x] Tick this box to confirm that a figure exemplifying the gating strategy is provided in the Supplementary Information.
